# Supplementary material for: Comprehensive prediction of secondary metabolite structure and biological activity from microbial genome sequences
Source: Nat Commun. 2020 Nov 27;11:6058. doi: 10.1038/s41467-020-19986-1 (PMC7699628; doi:10.1038/s41467-020-19986-1)
Supplement: Supplementary file 1 — Supplementary Information [file 41467_2020_19986_MOESM1_ESM.pdf]

Supplementary information

Comprehensive prediction of secondary metabolite structure and biological activity from microbial genome sequences

Michael A. Skinnider[1,2,3,4*], Chad W. Johnston[1,2,3,5], Mathusan Gunabalasingam[1,2], Nishanth J. Merwin[1,2], Agata M. Kieliszek[3], Robyn J. MacLellan[3], Haoxin Li[3], Michael R.M. Ranieri[1,2], Andrew L.H. Webster[1,2], My P.T. Cao[1,2], Annabelle Pfeifle[3], Norman Spencer[3], Q. Huy To[1,2], Dan Peter Wallace[3], Chris A. Dejong[3*], Nathan A. Magarvey[1,2]

[1] Department of Biochemistry & Biomedical Sciences, Michael G. DeGroote Institute for Infectious Disease Research, McMaster University, Hamilton, ON, Canada
[2] Department of Chemistry & Chemical Biology, Michael G. DeGroote Institute for Infectious Disease Research, McMaster University, Hamilton, ON, Canada
[3] Adapsyn Bioscience, Hamilton, ON, Canada
[4] Michael Smith Laboratories, University of British Columbia, Vancouver, BC, Canada
[5] Institute for Medical Engineering and Science, Massachusetts Institute of Technology, Cambridge, MA, USA
* e-mail: michaelskinnider@gmail.com, chris_dejong@adapsyn.com

Contents:

- Supplementary Figures 1–11
- Supplementary Tables 1–4
- Supplementary Note 1

Supplementary figures

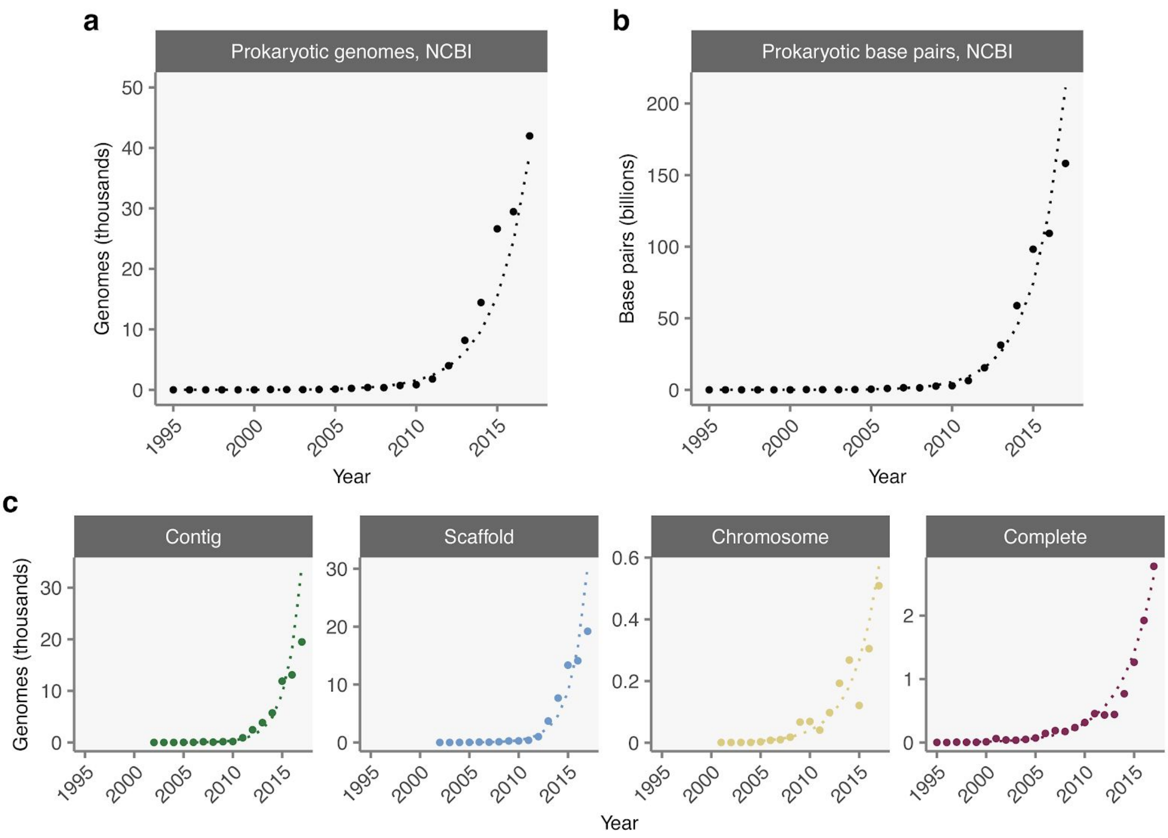

Supplementary Fig. 1 | Exponential accumulation of prokaryotic sequence information deposited in public databases. a, Total number of prokaryotic genome sequences deposited in the NCBI Genome database by year (1995–2017). Dashed lines show exponential fit. **b**, Base pairs of prokaryotic genome sequence deposited in the NCBI Genome database by year. **c**, Number of prokaryotic genome sequences deposited in the NCBI Genome database by year, subset by genome assembly level.

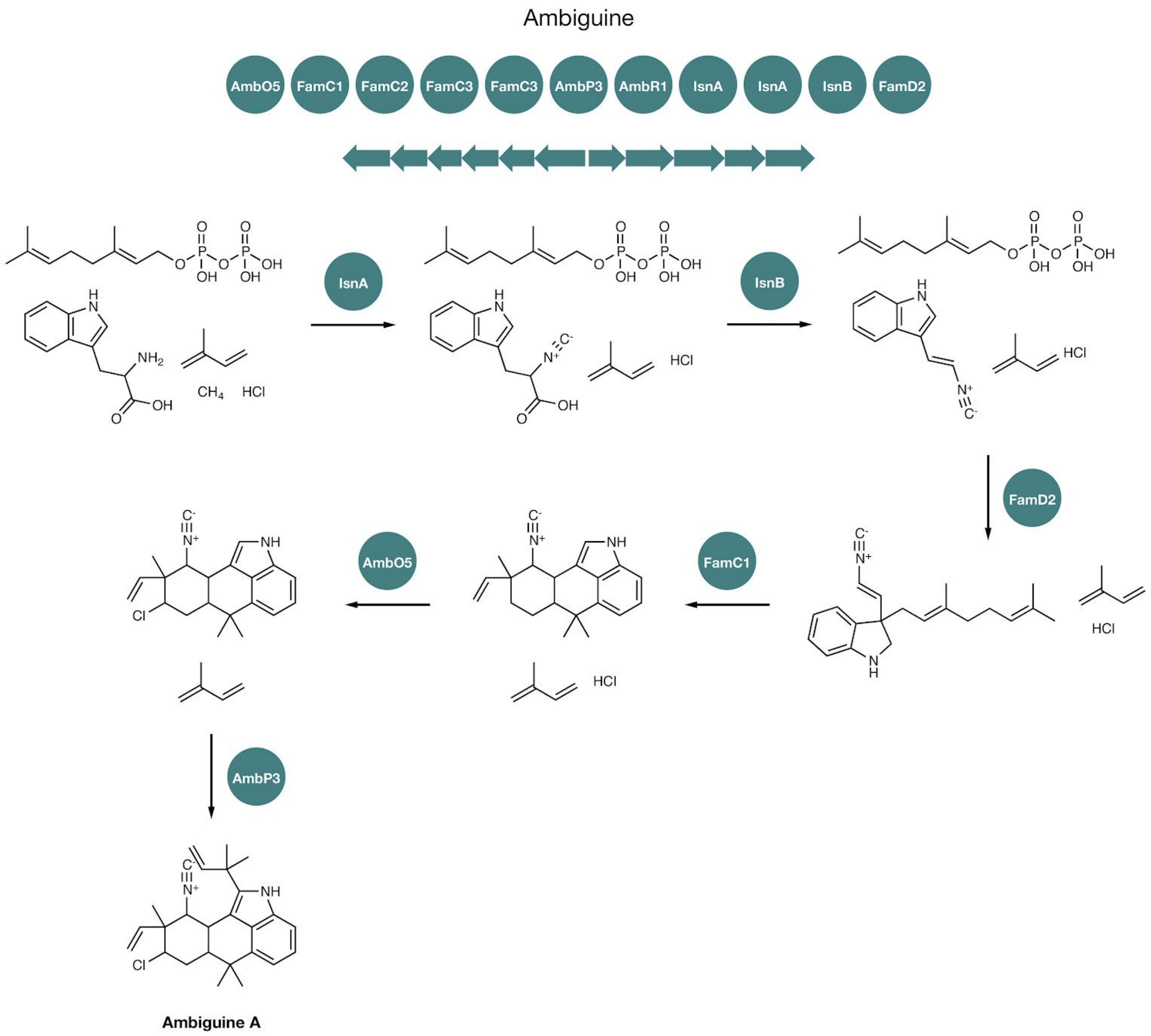

Supplementary Fig. 2 | *In silico* biosynthetic pathway inference by PRISM 4 within the ambiguine A biosynthetic gene cluster. PRISM 4 infers complete biosynthetic pathway for an arbitrary set of identified biosynthetic domains, based on a library of virtual tailoring reactions linking each biosynthetic enzyme to the chemical reactions it catalyzes. The biosynthetic pathway used internally within PRISM 4 to construct complete chemical structures, and output in JSON format from the PRISM 4 web server, is shown for ambiguine A.

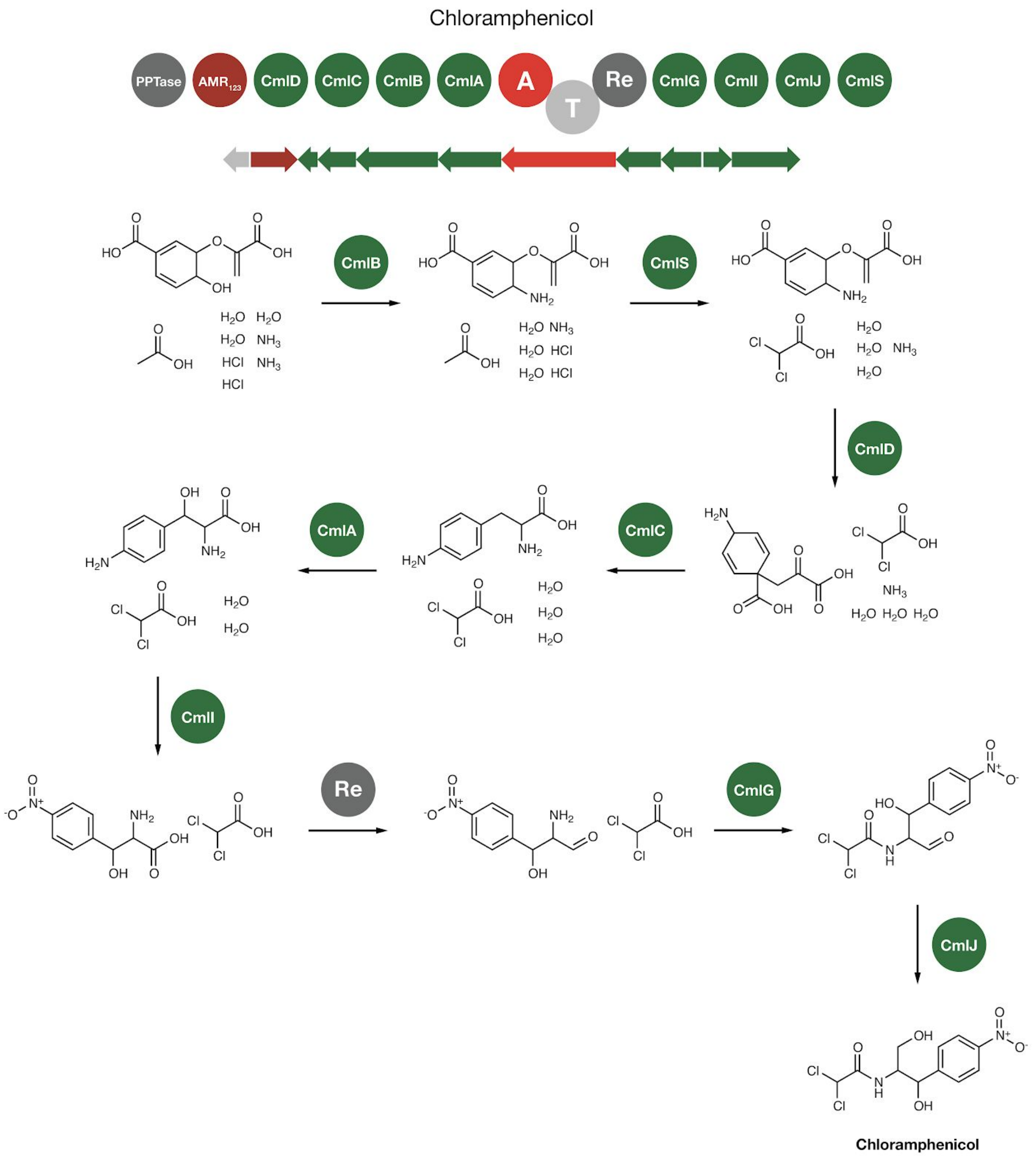

Supplementary Fig. 3 | *In silico* biosynthetic pathway inference by PRISM 4 within the chloramphenicol biosynthetic gene cluster. PRISM 4 infers complete biosynthetic pathway for an arbitrary set of identified biosynthetic domains, based on a library of virtual tailoring reactions linking each biosynthetic enzyme to the chemical reactions it catalyzes. The biosynthetic pathway used internally within PRISM 4 to construct complete chemical structures, and output in JSON format from the PRISM 4 web server, is shown for chloramphenicol.

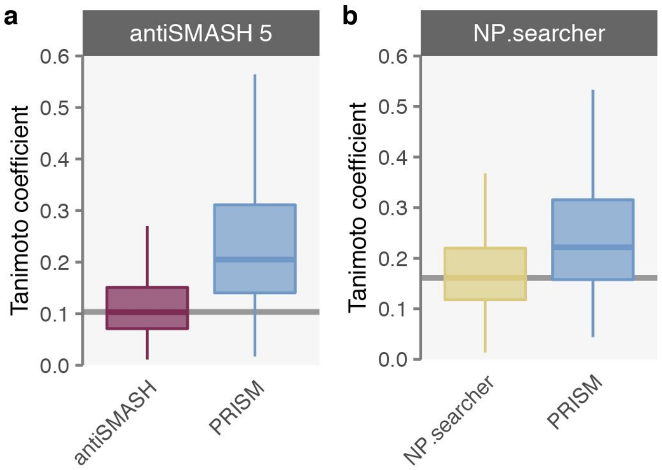

Supplementary Fig. 4 | Pairwise comparisons of structure prediction accuracy. Median Tanimoto coefficients between true and predicted structures for the subset of gold standard BGCs with at least one predicted structure generated by both PRISM 4 and antiSMASH 5 (**a**, n = 753) or PRISM 4 and NP.searcher (**b**, n = 398). Box plots show median (horizontal line), interquartile range (hinges), and the smallest and largest values no more than 1.5 times the interquartile range (whiskers) throughout.

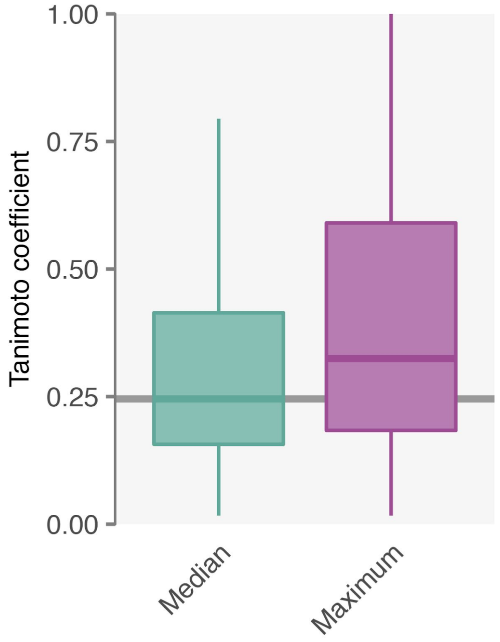

Supplementary Fig. 5 | Validation of combinatorial structure prediction in PRISM 4. Median and maximum Tanimoto coefficients between true and predicted structures generated by PRISM 4 for the gold standard set (n = 1,157). Box plots show median (horizontal line), interquartile range (hinges), and the smallest and largest values no more than 1.5 times the interquartile range (whiskers) throughout.

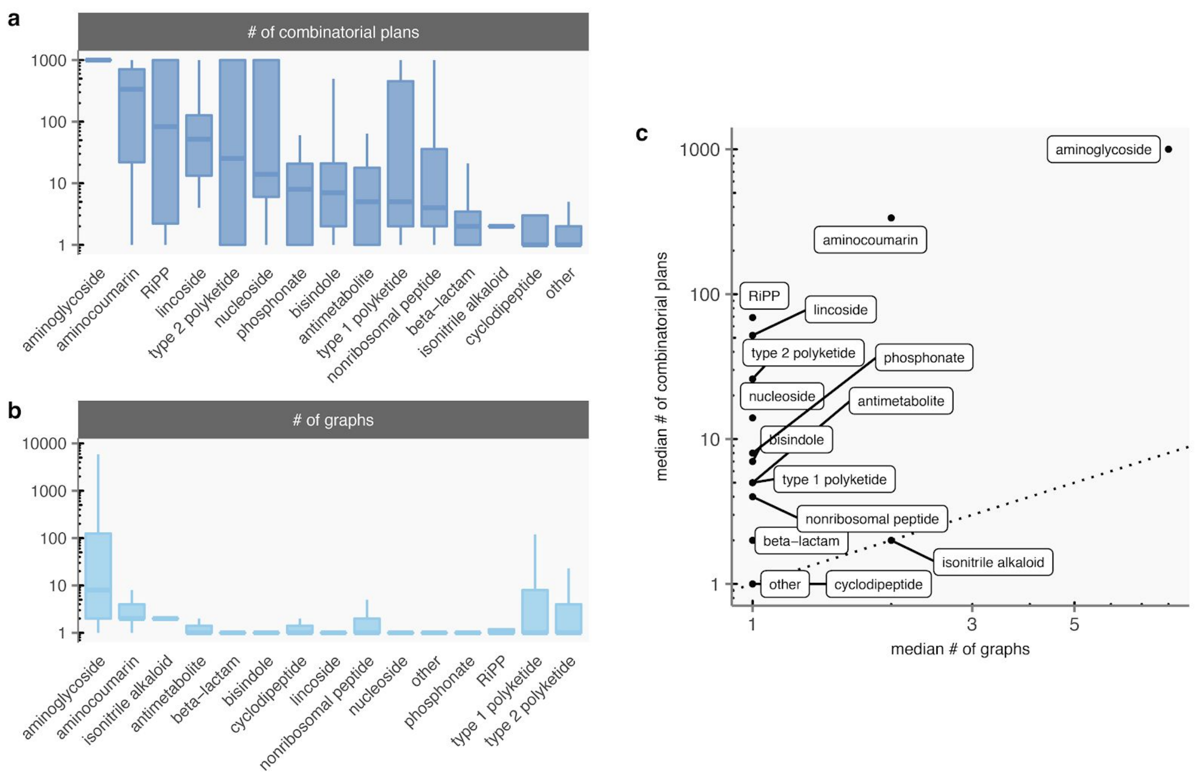

Supplementary Fig. 6 | Structural uncertainty and combinatorial search space of PRISM 4 chemical structure predictions. a–b, Number of combinatorial plans (**a**) and chemical graphs (**b**) considered by PRISM 4, for *n* = 1,157 BGCs with at least one predicted structure, grouped by biosynthetic family. **c,** Relationship between median number of combinatorial plans and chemical graphs for each biosynthetic family (dotted line shows the line y = x). Box plots show median (horizontal line), interquartile range (hinges), and the smallest and largest values no more than 1.5 times the interquartile range (whiskers) throughout.

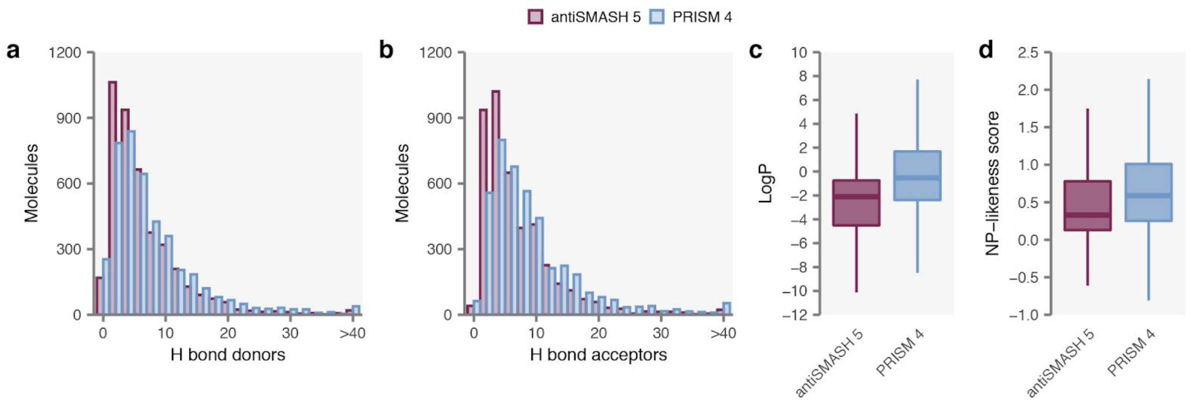

Supplementary Fig. 7 | Additional structural features of *n* = 4,220 pairs of predicted secondary metabolites from BGCs with products predicted by both PRISM 4 and antiSMASH. a–b, Number of hydrogen bond donors (**a**) and acceptors (**b**) in predicted chemical structures generated by PRISM 4 or antiSMASH 5. **c**, Octanol-water partition coefficients of predicted structures. **d**, Natural product-likeness scores of predicted structures. Box plots show median (horizontal line), interquartile range (hinges), and the smallest and largest values no more than 1.5 times the interquartile range (whiskers) throughout.

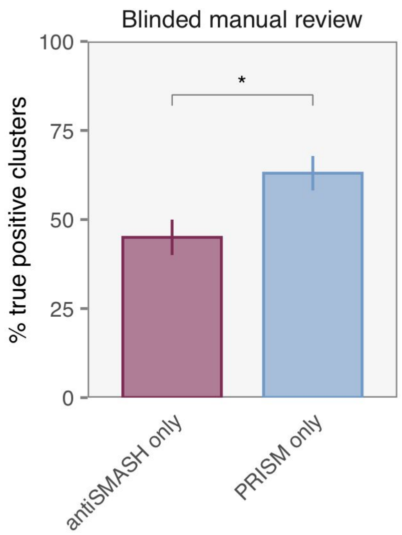

Supplementary Fig. 8 | Blinded manual review of BGC detection in PRISM 4 and antiSMASH 5. The proportion of true-positive clusters among two sets of 100 BGCs detected only by antiSMASH 5 and 100 BGCs detected only by PRISM 4, respectively, are shown. Error bars show the standard error of the sample proportion. *, p = 0.016, χ^2 test.

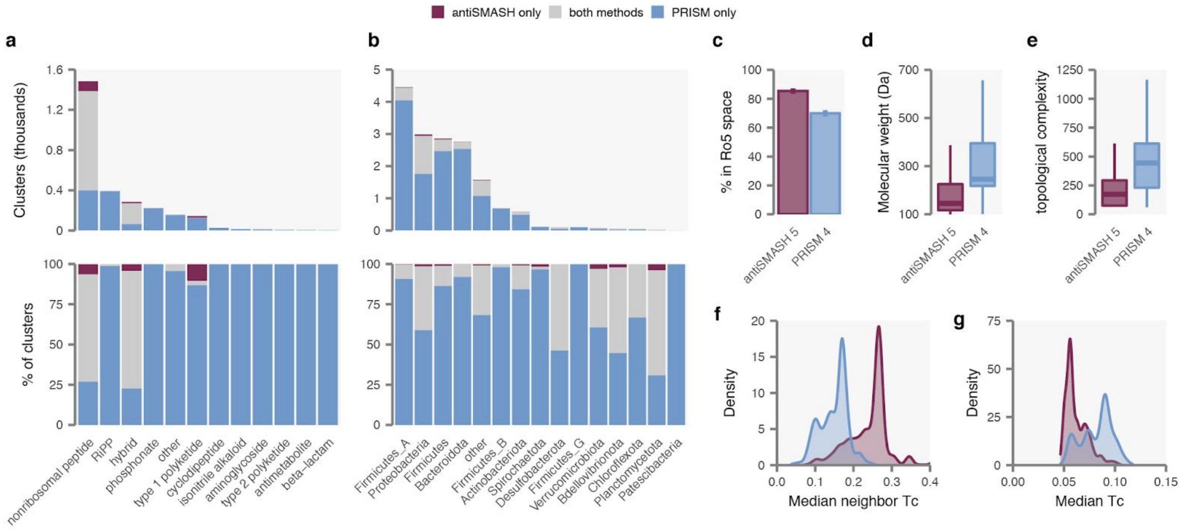

Supplementary Fig. 9 | PRISM 4 reveals secondary metabolite biosynthesis in 6,362 bacterial metagenome-assembled genomes (MAGs). a–b, Number of BGCs with at least one chemical structure predicted by PRISM 4, antiSMASH, or both methods in a collection of 6,362 dereplicated MAGs. **c–g,** Structural features of 1,212 pairs of predicted secondary metabolites from BGCs with products predicted by both PRISM 4 and antiSMASH 5. **c**, Percent of predicted structures in Lipinski rule of five space. Error bars show the standard error of the sample proportion. **d**, Molecular weight of predicted structures. **e**, Bertz topological complexity index of predicted structures. **f**, Internal diversity of predicted structures, as quantified by median Tanimoto coefficient to all other predicted structures in the set. **g**, Similarity of predicted structures to known natural products, as quantified by the median Tanimoto coefficient to the set of known natural products in the Natural Products Atlas. Box plots show median (horizontal line), interquartile range (hinges), and the smallest and largest values no more than 1.5 times the interquartile range (whiskers) throughout.

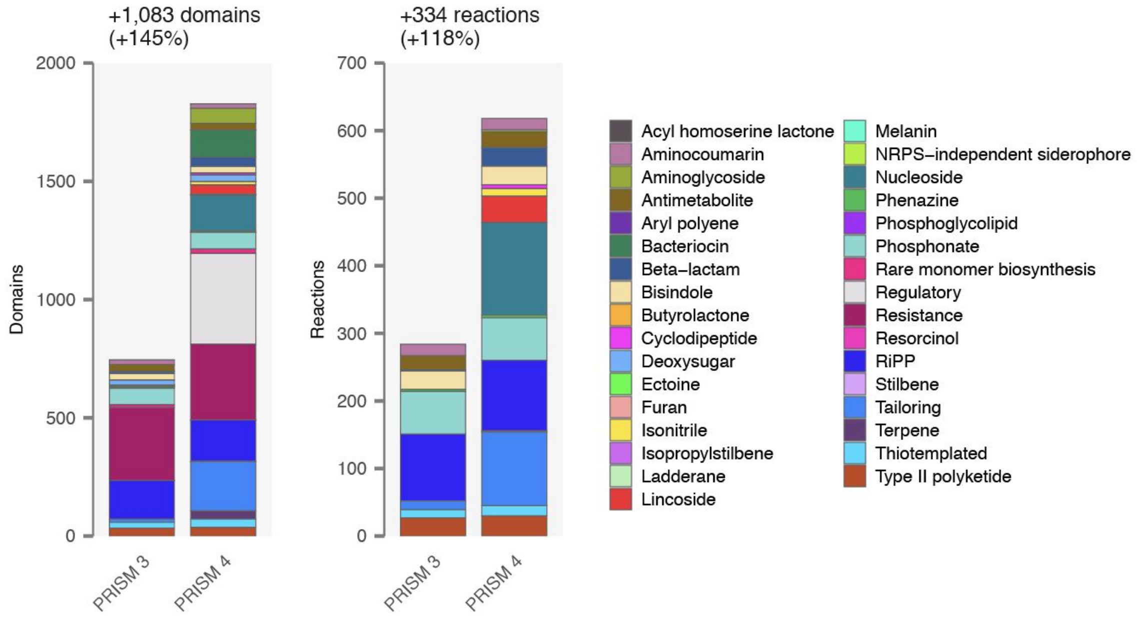

Supplementary Fig. 10 | Comparison of PRISM 3 and PRISM 4. Number of biosynthetic domains, left, and virtual tailoring reactions, right, incorporated in PRISM 4 and the previous release, PRISM 3.

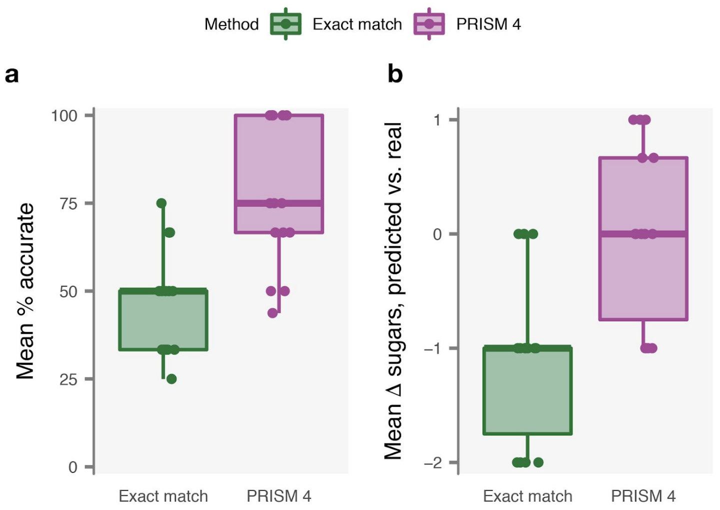

Supplementary Fig. 11 | Validation of aminoglycoside sugar prediction in PRISM. a, Mean proportion of correctly predicted sugars within known aminoglycoside clusters using the PRISM 4 method or a naive comparison requiring an exact match to known biosynthetic cassettes. **b**, Difference between true and predicted numbers of sugar or aminocyclitol moieties within known aminoglycoside clusters using the PRISM 4 method or a naive comparison requiring an exact match to known biosynthetic cassettes. Box plots show median (horizontal line), interquartile range (hinges), and the smallest and largest values no more than 1.5 times the interquartile range (whiskers) throughout. Panels **a-b** show data for n = 14 aminoglycoside BGCs.

Supplementary tables

Supplementary Table 1
Biosynthetic classes of secondary metabolites for which biosynthetic gene cluster detection and structure prediction is supported in PRISM 4.

Supplementary Table 2
Rare monomer biosynthesis domains whose presence in a biosynthetic gene cluster is required to permit prediction of certain rare monomers as adenylation, acyl-adenylating, or acyltransferase domain substrates using substrate-specific hidden Markov model libraries in PRISM 4.

Supplementary Table 3
Rules for biosynthetic gene cluster detection in PRISM 4. Hidden Markov model hits required to identify a biosynthetic gene cluster of the given type or subtype are listed. Rules for cluster types in bold indicate generic rules for detection of a cluster of that class, whereas more specific rules are included for the detection of several subtypes for some biosynthetic classes. Some rules for detection of particular cluster subtypes refer to other subtype rules, which are underlined.

Supplementary Table 4
Database of curated aminoglycoside biosynthesis pathways in PRISM 4.

Supplementary Table 1
Biosynthetic classes of secondary metabolites for which complete chemical structure prediction, or biosynthetic gene cluster detection (but not structure prediction), is supported in PRISM 4.

**Complete chemical structure prediction**
Aminocoumarins
Aminoglycosides
Antimetabolites
Beta-lactams
Bisindoles
Cyclodipeptides
Deoxy sugars
Isonitrile alkaloids
Lincosides
Nonribosomal peptides
Nucleosides
Phenazines
Phosphonates
RiPPs (25 families)
Stilbenes
Type I polyketides (except enediynes and iterative type I polyketides)
Type II polyketides
**Biosynthetic gene cluster identification only**
Acyl homoserine lactones
Aryl polyenes
Bacteriocins
Butyrolactones
Ectoines
Furans
Ladderanes
Melanins
NRPS-independent siderophores
Phosphoglycolipids
Resorcinols
**Other gene detection functionality**
Tailoring domains
Rare monomer biosynthesis domains
Regulatory domains
Resistance domains

Supplementary Table 2
Rare monomer biosynthesis domains whose presence in a biosynthetic gene cluster is required to permit prediction of certain rare monomers as adenylation, acyl-adenylating, or acyltransferase domain substrates using substrate-specific hidden Markov model libraries in PRISM 4.

Domain	Substrate (abbreviation)	Required biosynthetic domain(s) (abbreviation)
Adenylation	Beta-phenylalanine (β-Phe)	Phenylalanine ammonia lyase (PAL)
Adenylation	Beta-phenylalanine (β-Phe)	Phenylalanine 2,3-amino mutase (HitA)
Adenylation	Anthranilic acid activation (2AA)	Reductase, imine-forming variant (Re)
Adenylation	3-aminobutyric acid (Abu)	β-glutamate decarboxylase (IdnL3)<br>Glutamate 2,3-aminomutase (IdnL4)
Adenylation	2-amino-3,5-dimethylhex-4-enoic acid (ADH)	Isobutyryl-CoA dehydrogenase (CymE)<br>Isobutyrylaldehyde aldolase (CymF)<br>C-methyltransferase (CymG)<br>2-keto-4-hydroxy-isoheptanate hydratase (CymH)
Adenylation	Capreomycidine (Cap)	L-arginine hydroxylase (CmnC)<br>Capreomycidine synthase (CmnD)
Adenylation	2-carboxy-6-hydroxyoctahydroindole (Choi)	2,3'-dicarboxy-6-ketohexahydroindole decarboxylase (AerD)<br>Amino-prephenate cyclase (AerE)<br>2-carboxy-6-ketohexahydroindole dehydratase (AerF)
Adenylation	(2S,6R)-diamino-(5R,7)-dihydroxy-heptanoic acid (DADH)	Transketolase (Vzb27)<br>Transketolase (Vzb28)
Adenylation	2,3-diaminopropionate (Dap)	Diaminopropionate synthase (CmnB)
Adenylation	Enduracididine (End)	Arginine γ-monooxygenase/transaminase (MppP)<br>Enduracididine transaminase (MppQ)<br>α-keto-enduracididine cyclase (MppR)
Adenylation	Glycolic acid (GA)	D-xylulose 5-phosphate dehydrogenase, α subunit (Qcn7)<br>D-xylulose 5-phosphate dehydrogenase, β subunit
Adenylation	Homotyrosine (Hty)	Benzylmalate synthase (HphA)<br>Benzylmalate isomerase (HphB)<br>Benzylmalate isomerase (HphCD)
Adenylation	6-chloro-4-hydroxyindole-3-carboxylic acid (Ind)	6-chloro-tryptophan 4- and β-dihydroxylase (FmoC)<br>6-chloro-4-hydroxyindole aldehyde dehydrogenase (FmoI)<br>6-chloro-4-beta-dehydroxytryptophan aldolase (FmoM)

Adenylation	4-methylproline (MePro)	Methylproline cyclase (NosE)<br>Methylproline reductase (NosF)
Adenylation	2-methylserine (MeSer)	α-methyl serine synthase (AmiS)
Adenylation	Modified tyrosine (mTyr)	3-methyl-L-tyrosine peroxygenase (SfmD)<br>Tyrosine 3-C-methyltransferase (SfmM2)
Adenylation	Para-aminobenzoic acid (pABA)	Chorismate transaminase (CmlB)
Adenylation	Para-aminohydroxybenzoic acid (pAHBA)	Chorismate transaminase (CmlB)<br>Para-aminobenzoate meta-hydroxylase (Alb12)
Adenylation	Pipecolic acid (Pip)	Cyclodeaminase (GetD)<br>Hydroxylase (GetF)
Adenylation	Piperazic acid (Piz)	Piperazic acid synthase (KtzT)
Adenylation	Tambroline (Tam)	Tambroline lysine dehydrogenase (TbrP)<br>Tambroline cyclase (TbrQ)
Adenylation	Alanine activation (A)	Amine-deprotecting peptidase (VinJ)
Adenylation	Methylserine activation (A)	α-methyl serine synthase (AmiS)
Adenylation	Para-aminophenylalanine activation (A)	4-amino-4-deoxyprephenate dehydrogenase dehydrogenase (CmlC)
Adenylation	Proline activation (A)	Tyrosine ortho-hydroxylase (LmbB2)<br>Reductase, imine-forming variant (Re)
Acyl-adenylating	Para-hydroxybenzoic acid (pHBA)	Para-hydroxybenzoate CoA-ligase (Alb07)<br>Chorismate-pyruvate lyase (Alb20)
Acyltransferase	Methoxymalonate (OMeMal)	3-hydroxyacyl-CoA dehydrogenase (HADH)<br>Acyl-CoA dehydrogenase (ADH)

Supplementary Table 3
Rules for biosynthetic gene cluster detection in PRISM 4. Hidden Markov model hits required to identify a biosynthetic gene cluster of the given type or subtype are listed. Rules for cluster types in bold indicate generic rules for detection of a cluster of that class, whereas more specific rules are included for the detection of several subtypes for some biosynthetic classes. Some rules for detection of particular cluster subtypes refer to other subtype rules, which are denoted by underlines.

**Cluster type**	**Rules for cluster detection**
**Acyl homoserine lactone**	LasI
**Aminocoumarin**	Adenylation *and* NovI *and* NovJ *and* NovK
Aminocoumarin (rubradirin-type)	Adenylation *and* NovI *and* RubC3
**Aminoglycoside**	(IPM *or* 4,6DH *or* s2DOI) *and* ≥ 3 aminoglycoside genes
2-deoxy-streptamine derived aminoglycoside	DOI AmT *and* DOIA DH
Neamine- or paromamine derived aminoglycoside	2-deoxy-streptamine derived *and* 2NAPd
Kanamycin family aminoglycoside	Neamine- or paromamine derived *and* KanE
Neomycin/ribostamycin family aminoglycoside	Neamine- or paromamine derived *and* NeoF
Butirosin family aminoglycisode	Neamine- or paromamine derived *and* BtrG
Paromomycin family aminoglycoside	Neomycin/ribostamycin family *and* ParK
Lividomycin family aminoglycoside	Paromomycin family *and* LivW
Tobramycin family aminoglycoside	Kanamycin family *and* TobZ
Gentamicin family aminoglycoside	Neamine- or paromamine derived *and* GenD1
Fortimicin family aminoglycoside	Neamine- or paromamine derived *and* ForX
Istamycin family aminoglycoside	Fortimicin family *and* ImrA
Apramycin family aminoglycoside	Neamine- or paromamine derived *and* LivW *and* ImrA *and* AprH
Scyllo-inosose derived aminoglycoside	IPM *and* StrI
D- or L-myo-inosose derived aminoglycoside	IPM *and* SpcB
Kasugamycin family aminoglycoside	IPM *and* KasQ
**Aryl polyene**	APE_AT
**Antimetabolite**	
3-methylarginine	MrsA *and* MrsB
Anticapsin	BacA *and* BacB *and* BacC *and* BacF *and* BacG
Bacilysin	Anticapsin *and* BacD
Cycloserine	DcsA *and* DcsB *and* DcsD *and* DcsG
Dapdiamide	DdaC *and* DdaF *and* DdaG *and* DdaH
Indolmycin	Ind1 *and* Ind2 *and* Ind3

**Bacteriocin**	≥ 1 bacteriocin gene
Class II/III confident bacteriocin	<u>Bacteriocin</u> *and* (≥ 1 regulatory gene *and* ≥ 1 resistance gene)
**Beta-lactam**	
Simple carbapenem	CarA *and* CarB *and* CarC
Complex carbapenem	CarA *and* CarB *and* ThnT
Clavaminic acid	CEAS *and* BLS
5S clavam	<u>Clavaminic acid</u> *and* Cvm1
Clavulanic acid	<u>Clavaminic acid</u> *and* GCAS
Sulfazecin	Adenylation *and* Thiolation *and* Monobactam_TE
Tabtoxin	TblS
Nocardicin	NocJ *and* Adenylation
Penicillin	IPNS *and* Adenylation
Cephamycin	<u>Penicillin</u> *and* CmcH
Cephalosporin	<u>Penicillin</u> *and* CefEF
**Bisindole**	RebO *and* RebD
Bisindolylmaleimide	<u>Bisindole</u> *and* MarC
Carboxy indolocarbazole	<u>Bisindole</u> *and* EspM
Indolotryptoline	<u>Bisindole</u> *and* ClaX1
Maleimide indolocarbazole	<u>Bisindole</u> *and* RebC
Pyrrolinone indolocarbazole	<u>Bisindole</u> *and* StaC
Pyrrolinium indolocarbazole	<u>Bisindole</u> *and* RedE
Violacein	<u>Bisindole</u> *and* VioE
**Butyrolactone**	AfsA
**Cyclodipeptide**	CDPS
NYH family cyclodipeptide	CPDS (NYH subtype)
XYP family cyclodipeptide	CPDS (XYP subtype)
SYQ family cyclodipeptide	CPDS (SYQ subtype)
**Ectoine**	Ectoine_synthase
**Furan**	MmyO
**Isonitrile alkaloid**	IsnA *and* IsnB
**Isopropylstilbene**	StlC *and* StlD
**Ladderane**	LadDH *and* LadMT

**Lincoside**	LmbT *and* (LmbF *or* CcbF)
Alkylproline-containing	LmbA *and* (LmbW *or* TomN)
**Melanin**	MelC2
**NRPS-independent siderophore**	NISa *or* NISb *or* NISc
**Nucleoside**	
Amicetin-type nucleoside	AmiF *and* AmiJ
Blasticidin-type nucleoside	BlsD *and* MilG *and* ArgK
Mildiomycin-type nucleoside	BlsD *and* MilG *and* MilN
Gougerotin-type nucleoside	BlsD *and* GouJ
Jawsamycin-type nucleoside	Jaw2 *and* Jaw5
Glycyluridine-derived nucleoside	LipK *and* LipL
Capuramycin-type nucleoside	LipK *and* CapG *and* CapW
Liposidomycin-type nucleoside	LipK *and* LipP *and* Mur24 *and* LipW
Muramycin-type nucleoside	LipK *and* LipP *and* Mur24 *and* Mur23 *and* Mur30
Pacidamycin-type nucleoside	Pac5 *and* Pac9
Octosyl acid-derived nucleoside	PolA *and* PolH
Nikkomycin-type nucleoside	PolH *and* PolCDK *and* PolG *and* NikD
Polyoxin-type nucleoside	PolH *and* PolCDK *and* PolG *and* PolM
Toyocamycin-type nucleoside	(ToyH *or* TubE) *and not* QueF
Sangivamycin-type nucleoside	Toyocamycin-type *and* (ToyJ *or* ToyK *or* ToyL)
Ascamycin-type nucleoside	(AcmG *or* AcmI *or* AcmK) *and* AcmN
C-nucleoside	SdmA *and* SdmB
Puromycin-type nucleoside	Pur4 *and* Pur5 *and* Pur10
Tunicamycin-type nucleoside	TunB *and* TunF
A201A-type nucleoside	Hyg14 *and* MtdL
Hygromycin A-type nucleoside	A-201A-type *and* Hyg7
**Phenazine**	PhzAB *and* PhzD *and* PhzE
**Phosphoglycolipid**	MoeO5

**Phosphonate**	PEPM *and* (VlpB *or* FbrC *or* (RhiE *and* RhiF) *or* DhpF)
APPA-derived	PEPM *and* RhiG *and* RhiI *and* RhiJ
Argolaphos-type	PEPM *and* FomC *and* PhpD *and* PhpE *and* Arg10
Dehydrophos-type	PEPM *and* FomC *and* DhpA *and* DhpB *and* DhpC *and* DhpD *and* DphH_NTD
Fosfazinomycin-type	PEPM *and* DhpF *and* FzmA *and* FzmB *and* FzmF *and* FzmG *and* FzmL *and* FzmM *and* FzmN *and* FzmO
Fosfomycin-type	PEPM *and* Fom3 *and* Fom4 *and* FomC *and* (FrbC *or* DhpF)
FR-900098-type	PEPM *and* FrbA *and* FrbB *and* FrbE *and* FrbF *and* FrbG *and* FrbH *and* FrbI
Nitrilaphos-type	PEPM *and* AEP_AMT *and* NitB *and* NitC *and* NitD *and* NitE
Phosphine-containing	PEPM *and* PhpI
Valinophos-type	PEPM *and* VlpB
**Resorcinol**	DarB
**Ribosomally synthesized and posttranslationally modified peptide (RiPP)**	
Autoinducing peptide	AgrB *and* AgrD
Bacterial head-to-tail cyclized peptide	DUF95 *and* HTT_precursor
Bottromycin	BotA *and* BotC
ComX	ComQ *and* ComX
Cyanobactin	PatA *and* (PatG *or* PatG_ox)
Glycocin	SunA *and* SunS
Class I lantipeptide	LanB *and* LanC
Class II lantipeptide	LanM
Class III/IV lantipeptide	LanKC
Prochlorosin	ProcA
Lasso peptide	CapB *and* CapC
Linaridin	(CypA *or* LegA) *and* (CypH *or* LegH) *and* CypL
Linear azol(in)e-containing peptide	(McbB *and* (McbC *or* McbD)) *or* GodG
Microviridin	MdnA *and* (MdnB *or* MdnC)
Proteusin	PoyA *and* PoyD
Sactipeptide	SboA *and* AlbA
Streptide	StrA *and* StrB *and* StrC
Thiopeptide	LazB *and* LazC
Trifolitoxin-family peptide	TfxA *and* TfxB *and* TfxC
Thioviridamide-family peptide	TvaA *and* TvaH
YM-216391-family peptide	YmA *and* YmF
Pantocin	PaaA *and* PaaB

Phosphoramidate	MccB *and* MccC
α-keto-β-amino acid-containing peptide	PlpX
**Thiotemplated**	(Condensation *or* Ketosynthase) *and* (Adenylation *or* Acyltransferase *or* Acyl-adenylating)
Polyketide	<u>Thiotemplated</u> *and* ≥ 1 complete polyketide module
Nonribosomal peptide	<u>Thiotemplated</u> *and* ≥ 1 complete nonribosomal peptide module
Enediyne (9-membered)	<u>Thiotemplated</u> *and* PPTase *and* 9-membered enediyne ketosynthase
Enediyne (10-membered)	<u>Thiotemplated</u> *and* PPTase *and* 10-membered enediyne ketosynthase
Iterative type I polyketide	Ketosynthase *and* (PT_I *or* PT_II *or* PT_III *or* PT_IV *or* PT_V)
Chloramphenicol	CmlC *and* CmlG
**Type II polyketide**	KSa *and* CLF
Tetracycline-type polyketide	<u>Type II polyketide</u> *and* tetracycline-type CLF
Anthracycline-type polyketide	<u>Type II polyketide</u> *and* anthracycline-type CLF
Angucycline-type polyketide	<u>Type II polyketide</u> *and* angucycline-type CLF
Aureolic acid polyketide	<u>Type II polyketide</u> *and* aureolic acid-type CLF
Tetracenomycin-type polyketide	<u>Type II polyketide</u> *and* tetracenomycin-type CLF
Benzoisochromanequinone polyketide	<u>Type II polyketide</u> *and* benzoisochromanequinone-type CLF
Pentangular polyphenol	<u>Type II polyketide</u> *and* pentangular polyphenol-type CLF
Pluramycin-type polyketide	<u>Type II polyketide</u> *and* pluramycin-type CLF
Resistomycin-type polyketide	<u>Type II polyketide</u> *and* resistomycin-type CLF
Enterocin-type polyketide	<u>Type II polyketide</u> *and* enterocin-type CLF

Supplementary Table 4
Database of annotated biosynthetic pathways involved in aminocyclitol and amino sugar biosynthesis in PRISM 4.

Aminocyclitol/ amino sugar	Chemical structure (SMILES)	Biosynthetic domains
2-deoxystreptamine	NC1CC(N)C(O)C(O)C1O	2DOI synthase (s2DOI)<br>LGln 2DOI aminotransferase (DOI AmT)<br>ForE dehydrogenase (DH)<br>ForB 6' aminotransferase (NMT)<br>ForO O-methyltransferase (OMT)
3-deoxyneosamine C	NCC1OC(O)C(N)CC1O	Deacetylase (DeAc)<br>Paromamine C6 dehydrogenase (DH)<br>Paromamine C6 aminotransferase (AmT)<br>Neamine C3 oxidoreductase (Ox)<br>Neamine C3 dehydrogenase (DH)<br>N-acetylglucosaminyl transferase (NGTr)
3,4-dideoxy purpurosamine	CC(N)C1CCC(N)C(O)O1	ForQ dehydrogenase (ForQ)<br>ForB 6' aminotransferase (AmT)<br>ForK C-methyltransferase (CMT)
Actinamine	CNC1C(O)C(O)C(O)C(NC)C1O	SpcA phosphatase (Phos)<br>SpcB dehydrogenase (DH)<br>SpcS2 aminotransferase (AmT)<br>SpcM N-methyltransferase (NMT)
Bluensidine	NC(=N)NC1C(O)C(O)C(O)C(NC(N)=O)C1O	Inositol phosphate monophosphatase (IPM)<br>StrI-family dehydrogenase (StrI)<br>StrB1 amidinotranserase (AmdTr)<br>Scyllo-inosose aminotransferase (StsC)
Dihydrostreptose	CC1OC(O)C(O)C1(O)CO	Aminoglycoside phosphotransferase (StrN)<br>dTDP-D-glucose synthase (StrD)<br>dTDP-D-glucose 4,6-dehydratase (StrE)<br>dTDP 4-keto-6-deoxy-D-glucose 3,5-epimerase (StrM)<br>dTDP-L-rhamnose synthase (StrL)
Fortamine	CNC1C(O)C(O)C(N)C(O)C1OC	Inositol phosphate monophosphatase (IPM)<br>dTDP-L-rhamnose synthase (StrL)<br>LGln 2DOI aminotransferase (DOI AmT)<br>ForE dehydrogenase (DH)<br>ForN N-methyltransferase (NMT)<br>ForO O-methyltransferase (OMT)
Inositol	OC1C(O)C(O)C(O)C(O)C1O	KasA inositol transferase (ITr)
Kanosamine	NC1C(O)C(O)OC(CO)C1O	KanC dehydrogenase (KanC)<br>KanD aminotransferase (KanD)<br>KanE-family glycosyltransferase (KanE)

Kasugamine	CC1OC(O)C(N)CC1NC(=N)C(O)=O	KasF acetyltransferase (AcT)<br>KasD 4,6 dehydratase (4,6DH)<br>KasR dehydratase (DH)<br>KasP reductase (Red)<br>KasC aminotransferase (AmT)<br>Glycine oxidase (Ox)
Myo-inositol	C1(C(C(C(C(C1O)O)O)O)O)O	Inositol phosphate monophosphatase (IPM)<br>2-epimerase (KasQ)
N-methyl-glucosamine	CNC1C(O)OC(CO)C(O)C1O	StrN phosphotransferase (PT)<br>StrQ cytidylyl transferase (CyTr)<br>StsB oxidoreductase (OxR1)<br>StrS aminotransferase (AmT)<br>StsG N-methyltransferase (NMT)
Streptidine	NC(N)=NC1C(O)C(O)C(O)C(N=C(N)N)C1O	Inositol phosphate monophosphatase (IPM)<br>StrI-family dehydrogenase (StrI)<br>StrB1 amidinotranserase (AmdTr)<br>StsB oxidoreductase (OxR1)<br>Scyllo-inosose aminotransferase (StsC)
Streptose	CC1OC(O)C(O)C1(O)C=O	Aminoglycoside phosphotransferase (StrN)<br>dTDP-D-glucose synthase (StrD)<br>dTDP-D-glucose 4,6-dehydratase (StrE)<br>dTDP 4-keto-6-deoxy-D-glucose 3,5-epimerase (StrM)<br>dTDP-L-rhamnose synthase (StrL)

Supplementary Note 1

PRISM 4 web application

Overview. PRISM 4 is a Java 7 web application, freely available as an online service for the research community at http://prism.adapsyn.com. The PRISM web application is powered by Vue.js with a lightweight Python Flask API using PostgreSQL and Redis for queue management, providing a scalable solution that can process many submissions at once. PRISM 4 implements the Chemistry Development Kit (version 1.4.19)[1] for chemical structure prediction and all *in silico* tailoring reactions, and BioJava (version 4.2.9)[2] for some sequence file input and output operations. Other Java library dependencies include Apache Batik, Apache Commons, and Apache HttpComponents; 'combinatoricslib' (version 2.0, available from https://github.com/dpaukov/combinatoricslib); and the Jackson JSON processing library. System dependencies include BLAST+ (version 2.2.30)[3] and HMMER (version 3.1b2)[4] for protein similarity search. Optional system dependencies include FIMO (version 4.11.1)[5], for RiPP precursor peptide cleavage[6]; Prodigal (version 2.6.1)[7], for prokaryotic ORF prediction; MUSCLE (version 3.8.31)[8], for active site residue identification in tRNA-derived cyclodipeptide synthases (CDPSs)[9]; and R (version 3.3.1), with packages 'class' (version 7.3-12), 'e1071' (version 1.6-7), and 'plyr' (version 1.8.4), for CDPS aminoacyl-tRNA substrate prediction[9].

User interface. The PRISM 4 user interface features 'one-click' submission of FASTA or GenBank format nucleotide sequence files, meaning no specialized computational training is required to interact with the web application. However, detailed instructions, including an interactive tutorial exploring an annotated series of outputs from an example genome, are also provided via the web application. A small number of advanced options are also available to users, including (i) the maximum number of predicted structures to generate for a given BGC (default: 50); (ii) the method or combination of methods to employ for open reading frame (ORF) prediction, which may be predicted using Prodigal[7], read from GenBank features, or identified *de novo* using a search of all potential protein-coding regions flanked by a start and stop codon (default: all three) (iii) the maximum window, in nucleotides, to consider when extending cluster boundaries (default: 10 kb); and (iv) the option to selectively enable or disable any of the 28 categories of biosynthetic domain search in PRISM 4. By default, all searches are carried out; enabling thiotemplated search, the default setting, subsequently triggers three additional searches, including type II polyketides, biosynthetic domains involved in the biosynthesis of rare monomers, and generic tailoring reactions not specific to any individual cluster type.

Output. PRISM generates rich interactive web pages including vector and HTML5-based graphics as output. Users are encouraged to explore the interactive tutorial, which provides an interactive, step-by-step walkthrough of an example output from the web application. Identified BGCs are colored by family and visualized on a 'virtual genome' in a circular layout, which delineates boundaries between contigs in the case of incomplete assemblies. Detailed information about each individual BGC is provided on a separate HTML page, which includes visualizations of the biosynthetic ORFs and the domains therein both as a linear sequence of ORFs, and as a conventional biosynthetic assembly graph. For each ORF, detailed information about the hidden Markov model (HMM) searches used to identify each domain, as well as any subsequent analysis (including predicted substrates, BLAST homology results, HMM-based domain subtype inference, RiPP propeptides, and active site residues), is displayed in a tabular format. Visualizations are also generated for each ORF sequence, demarcating the positions of predicted domains and active site residues along the linear

protein sequence. Domains are highlighted within the protein sequence itself to facilitate homology searches for domains of interest. Finally, for BGCs with predicted sugar moieties, including hexose, deoxy, and aminoglycoside sugars, an additional explanatory visualization is generated based on the detailed output from the PRISM sugar inference engine[10,11]. This visualization captures the biosynthetic logic underlying the predicted sugar combination(s), including the known biosynthetic pathway for each sugar and the domains within this pathway that were, or were not, identified within the cluster of interest. Predicted structures are output in SMILES format, in both tab-delimited and GNP-compatible[11] format, and can optionally be visualized in the browser in a modal slideshow interface, with rendering performed server-side by the RDKit (version 2016.03.5).

JSON output. In addition to the HTML-based interactive output, detailed output is provided in a machine-readable JSON format. JSON files for a given PRISM search can also be saved locally and re-opened later in the web application interface, without re-running the PRISM search, using the 'Open' application view. The JSON file includes some information not visually presented to the user within the interactive web application, most notably including the structures of intermediate products within predicted biosynthetic pathways (Supplementary Figs. 2 and 3).

Biosynthetic gene cluster detection in PRISM 4

ORF search. Contigs are read from the input FASTA or GenBank sequence file, and ambiguous IUPAC nucleotide codes (i.e., any of RYKMSWBDHV) are replaced by randomly sampling one of the possible nucleotides; all other characters are replaced with Ns. If more than 1,000 contigs are provided in a single file, only the 1,000 largest are retained for analysis. ORFs are subsequently identified within each contig separately, with overlapping ORFs handled by preferring GenBank annotations to Prodigal predictions, and the latter to potential protein-coding sequences. Prodigal is run in single-genome mode if the largest contig is at least 500 kb long, and metagenomic mode otherwise.

Domain search. Identified ORFs are searched with a library of 1,772 HMMs to identify protein domains linked to secondary metabolite biosynthesis (Supplementary Data 1). The identification of these domains provides the basis to predict *in silico* complete biosynthetic pathways in PRISM 4, based on the virtual tailoring reactions they are inferred to catalyze. For a subset of domains, further sequence characterization is performed by a combination of HMM- and BLAST-based methods, as follows. For substrate-activating domains from thiotemplated pathways (i.e., adenylation, acyl-adenylating, and acyltransferase domains), substrate prediction is performed by searching each domain with a library of 109, 27, or 15 substrate-specific HMMs, respectively, of which a total of 63 are derived from a previous study[12] and the remaining 88 were developed specifically for PRISM. The substrate associated with the top-ranked HMM is inferred to be activated by the domain in question. Many substrates are represented by more than one redundant model, in cases where patterns of sequence similarity supported the creation of multiple HMMs[12]. In other cases, HMMs are specific to clades of adenylation domains that activate an amino acid or starter unit, commonly as part of rare monomer biosynthesis, but are not directly associated with addition of a monomer to the growing scaffold. For a subset of HMMs associated with activation of 27 rare monomers, further supporting evidence is required to permit prediction of these substrates, in the form of the presence of biosynthetic domains associated with their biosynthesis within the BGC; these prerequisites are enumerated in Supplementary Table 2.

A second subset of domains are analyzed by BLAST searches against carefully curated databases. Condensation domains are searched against a database of 189 annotated condensation domain sequences from the NaPDoS database[13], which are used to identify condensation domains with epimerization (E), heterocyclization (Cyc), or starter unit-acylating (C*) functionalities[14]. Putative starter condensation domains identified by NaPDoS are subsequently searched by a second, more fine-grained database, consisting of an additional 41 starter condensation domain sequences, which is used to predict the likely chemical substrate involved in N-acylation by the condensation domain[11]. Ketosynthase domains are searched against a database of 1,371 annotated ketosynthase domain sequences, which is used to identify decarboxylative ketosynthases (KS_Q), inactive ketosynthases (KS_0), and iterative ketosynthases (KS_i), as well as ketosynthases involved in 9- or 10-membered enediyne biosynthesis (Gunabalasingam et al., unpublished results). Glycosyltransferase domains are searched against a database of 71 annotated glycosyltransferase domain sequences, which is used to discriminate between putative hexose and deoxy sugar glycosyltransferases, as described further below. Chlorinase domains are searched against a database of 32 natural product halogenases with annotated substrates, which is used to prioritize potential sites of chlorination reactions within predicted products[11]. Type II polyketide priming acyltransferase domains are searched against a database of 19 annotated sequences, which are used to classify priming acyltransferase domains activating propionate, butyrate, 2-methylbutyrate, hexadienoate, benzoate, or malonamate[11]. Finally, type II polyketide chain length factor (CLF) domains are searched against an annotated database of 69 CLFs, with the top hit used to infer the number of ketide chain extension cycles in the linear poly-β-ketide chain[11].

A third subset of domains are analyzed by libraries of subtype-specific HMMs, using a strategy similar to that used to identify adenylation, acyl-adenylating, and acyltransferase domain substrates. A set of three HMMs is used to assign CDPSs to one of three phylogenetic families (NYH, XYP, SYQ)[9,15]. Based on the observation that active and inactive modular polyketide reductive loop domains form distinct phylogenetic clades, we additionally developed libraries of 33 (27 active, 6 inactive) and 114 (88 active, 26 inactive) clade-specific HMMs to identify inactive ketoreductase and dehydratase domains, respectively (Gunabalasingam et al., unpublished results).

Finally, overlapping domains are removed, such that only the highest-scoring domain is retained, and only the top ten substrates and BLAST results for each domain are carried forward for further analysis and output.

Biosynthetic gene cluster identification. Following domain search and subtype assignments, BGCs are identified using a simple greedy algorithm in combination with a rule-based approach to BGC definition. Starting with any ORF containing at least one secondary metabolite biosynthesis domain (that is, any PRISM domain except resistance or regulatory domains), a user-defined window, defaulting to 10 kb is extended on either end of the ORF. If any further secondary metabolite domains are identified within this window, the window is extended again from the most distant domain. This process repeats iteratively until no more biosynthetic domains can be identified on either end of the candidate BGC.

Next, a rule-based approach is applied to determine whether to retain the candidate BGC for further analysis. Examples of these rules include the presence of the tryptophan amine oxidase RebO and the chromopyrrolic acid synthase RebD within the candidate BGC, required for bisindole identification, or the isonitrile synthase IsnA and and isonitrile acrylate synthase IsnB, required for isonitrile alkaloid identification. If multiple rules are satisfied for a single candidate BGC, the BGC is assigned multiple cluster types or subtypes. For example, the BGC for the antibiotic and herbicide

phosphinothricin contains enzymatic machinery associated with the biosynthesis of both nonribosomal peptides and phosphonate-containing secondary metabolites; it is therefore labelled a hybrid nonribosomal peptide/phosphonate BGC. Certain BGC families associated with both a generic detection rule as well as more specific rules for specific chemotypes within a given biosynthetic family. For example, bisindole BGCs that also contain the maleimide indolocarbazole epoxidase ClaX1 are labeled as indolotryptolines. Other families of BGCs have sufficiently diverse biosynthetic origins that only subtype-specific rules can be formulated. For example, the rule for identification of octosyl acid-derived nucleoside BGCs (requiring the presence of the UMP-enolpyruvyltransferase PolA and the phospho-octosyl acid synthase PolH) does not overlap at all with that for glycyluridine-containing nucleosides (requiring the uridine-5'-aldehyde transaldolase LipK and uridine-5'-monophosphate dioxygenase LipL). The complete table of rules is provided in Supplementary Table 3. If the candidate BGC does not satisfy any of the rules, it is discarded. Notably, the majority of these rules require two or more biosynthetic domains to be co-localized within a relatively small genomic window, reducing the likelihood of false positives relative to approaches based solely on individual marker genes[6].

Multi-domain biosynthetic module identification. The system for detection of thiotemplated BGCs (i.e., nonribosomal peptides and type I polyketides) within PRISM relies on the identification of not only individual domains, but also multi-domain biosynthetic modules within these megasynthetases. Each of these modules catalyzes the extension of the growing secondary metabolite scaffold by a single residue. In nonribosomal peptide synthetases (NRPSs), the canonical module consists of a linear sequence of condensation, adenylation, and thiolation (peptidyl carrier protein) or thioesterase domains, with the module possibly containing additional tailoring enzymes such as N-methyltransferases or nitroreductases. In polyketide synthases (PKS), the canonical module consists of a ketosynthase, acyltransferase, and thiolation (acyl carrier protein) or thioesterase domains, possibly also containing domains of the polyketide reductive loop (ketoreductase, dehydratase, enoylreductase) in addition to other tailoring enzymes such as O-methyltransferases or pyran synthases. Variants of these modules are also possible; for example, NRPS modules that begin biosynthesis may lack a condensation domain. In some cases, a single domain rather than a multi-domain module catalyzes the addition of a ketide, amino acid, fatty acid, or starter unit residue to the product of a thiotemplated BGC. Examples include decarboxylative ketosynthases (KS_Q)[16], β-branching domains in polyketide biosynthesis[17], Gcn5-related N-acetyltransferase loading domains[18], FkbH-like loading domains[19], starter condensation domains[14], amidotransferases involved in glutarimide biosynthesis[20], and acyl-adenylating domains[11]. These domains are also labeled as 'modules,' for the purposes of BGC detection.

Identification of complete, multi-domain modules has several advantages in the context of genome-guided chemical structure prediction, relative to approaches based solely on individual substrate-activating domains. Most notably, a large family of polyketides are defined by the absence of acyltransferase domains within their PKS modules, with this activity instead provided in *trans* by a single, standalone acyltransferase domain, or a small number thereof[21]. A smaller but nonetheless considerable number of trans-acting adenylation domains have also been reported within NRPSs. The identification of all possible NRPS or PKS modules within a BGC of interest renders the enumeration of all sites of *trans*-acyltransferase or adenylation domain activity straightforward. Second, some adenylation domains are involved in nonproteinogenic amino acid biosynthesis, but do not directly contribute to the extension of the growing scaffold; these are often found as standalone adenylation domains within a BGC[11]. By default, adenylation domains that are not part of an identifiable module are assumed to be biosynthetically inactive in PRISM, although there are a handful of exceptions to

this rule, most notably for standalone adenylation domains involved in macrolactam polyketide biosynthesis[22] and prolyl-AMP ligases involved in pyrrole biosynthesis[23]. Finally, to predict complete structures for the product of a BGC with multiple NRPS or PKS enzymes, it is necessary to infer the permutation in which those enzymes contribute to the maturation of the growing product, and therefore the order of the residues in the complete structure. The detection of multi-domain biosynthetic modules can assist in this inference, as described further below.

After the identification of modules is complete, a final series of checks is performed to ensure their biosynthetic plausibility prior to chemical structure prediction. Standalone prolyl-AMP ligases are inactivated if the BGC does not also contain a proline dehydrogenase domain to catalyze pyrrole biosynthesis. *Trans-acting* adenylation and acyltransferase domains are likewise inactivated if the BGC does not contain at least one *trans*-adenylation or *trans*-acyltransferase insertion module, respectively. When a BGC contains multiple starter unit-activating modules (i.e. starter condensation and acyl-adenylating domains), the starter condensation module(s) are inactivated. Finally, for type II polyketides, the activities of two types of domains that are paraphyletic with respect to their biochemical functions are inferred based on the remainder of the BGC. First, phylogenetic analysis of type II polyketide C-methyltransferases[24] has revealed that these assort based on their primary sequence according to the regioselectivity of the reaction they catalyze, with respect to position within the poly-β-ketide chain. A single exception to this trend is observed for homologs of the oxytetracycline C6 C-methyltransferases, which act at C8 in pentangular polyphenols. Thus, if a BGC contains a pentangular polyphenol cyclase, these methyltranferases are reassigned as C8 C-methyltransferases. Similarly, whereas the cyclization pattern catalyzed by type II polyketide cyclases is largely predictable from phylogenetic patterns of primary sequence conservation[25], a single polyphyletic clade includes both anthracyclines and tetracycline/aureolic acid fourth ring cyclases. For cyclases of this clade, the cyclization pattern is inferred based on the CLF within PRISM.

Chemical graph-based structure prediction in PRISM 4

Overview. The initial release of PRISM[11], designed specifically for nonribosomal peptide and polyketide structure prediction, modeled predicted secondary metabolites as linear permutations of monomers, each of which could be associated with one or more tailoring reactions. Although this framework mirrored the biochemistry of canonical thiotemplated pathways to a first approximation, and was successfully extended to model RiPP biosynthesis[6], it was not extensible to classes of secondary metabolites that cannot reasonably be considered as derivatized linear chains of monomers. With PRISM 3, we undertook a ground-up rewrite of the structure prediction framework in order to model secondary metabolites as chemical graphs[26]. Under this framework, individual enzymatic domains or groups thereof can be associated with the activation of chemical subgraphs, as well as the modification of existing subgraphs via enzymatic tailoring reactions. This graph-based framework affords a great deal of flexibility in modelling complete biosynthetic pathways and faithfully representing enzymatic transformations by permitting manipulation of biosynthetic intermediates at the level of individual bonds or atoms, rather than entire monomers. Here, we provide an overview of key aspects of the graph-based chemical structure prediction framework within PRISM 4, and its approach to combinatorial enumeration of all possible products, given the set of possible biosynthetic transformations associated with the enzymes at a given locus.

Chemical graph generation. The first step of combinatorial structure prediction within PRISM entails the generation of the complete set of chemical matter that will be involved in subsequent biosynthetic

transformations: that is, the *chemical graph*. Subsequent transformations of the chemical graph (that is, tailoring reactions) can modify or remove, but not add, chemical matter. Importantly, it may not be possible to unambiguously infer the set of chemical matter from the DNA sequence of the BGC alone. For instance, BGCs encoding RiPPs may contain multiple precursor peptides. Other BGCs may encode biosynthetic machinery for deoxy or amino sugar biosynthesis, but which may be consistent with multiple different sugar products. Thus, more than one chemical graph may be generated for any given BGC, up to some maximum.

A single chemical graph is composed of one or more *chemical subgraphs*. Each subgraph is associated with one or more domains, which are considered to have 'activated' that subgraph from primary metabolism within PRISM. Subgraphs themselves are composed of one or more *residues*. Subgraphs commonly consist of simple biosynthetic precursors from primary metabolism: for instance, nucleotides, shikimate pathway intermediates, or proteinogenic amino acids. Subgraphs may also be single atoms introduced by simple tailoring reactions, such as methyl, hydroxyl, or halogen groups. In these cases, a subgraph contains a single residue (e.g., a halogen atom or an alanine residue). However,e a subgraph may also comprise a combination of residues with a fixed pattern of connectivity: for instance, the chain of proteinogenic amino acids in a RiPP precursor peptide, or a linear poly-β-ketide chain in a type II polyketide. Biosynthetically rational permutations of residues activated by canonical thiotemplated machinery[11] (that is, nonribosomal synthetases and type I polyketide synthases) are also modeled as individual subgraphs.

Tailoring reaction enumeration. The next step in combinatorial structure prediction is the enumeration of all possible sites of each tailoring reaction, and all combinations thereof, for each chemical graph. This step relies on the division within PRISM of a single tailoring reaction into five component parts: a hidden Markov model, an *annotator class*, a *reaction class*, a *reaction priority*, and an indicator of whether the reaction can occur more than once.

The function of the *annotator* is to identify possible sites at which the tailoring reaction can occur. For example, the annotator for a proline dehydrogenase identifies all proline residues within the chemical graph. When the tailoring reaction is catalyzed by an enzyme that also introduces one or more subgraphs into the chemical graph, the annotator may include multiple subgraphs, or combinations thereof. For example, the annotator for a tryptophan dioxygenase will identify combinations of the oxygen atom activated by that enzyme with a single tryptophan residue. The output of an annotator is a *reaction plan*, which contains all the information necessary for the *reaction class* to execute the reaction. A reaction plan minimally consists of a list of atoms, each of which is associated with one or more *tags*. Tags can specify information related to the specific residue that has been activated (for example, 'γ carbon' or 'indole carbon 3'), which are automatically populated during the process of residue generation within PRISM. Tags can also specify information relevant to the tailoring reaction itself, such as 'methylation site' or 'pyridine residue 1 alpha carbon'. A reaction plan can also include arbitrary *annotations*, which capture information about the course of the reaction beyond tags: for instance, the chain of nucleophilic cyclization events catalyzed by a polyether epoxide hydrolase can terminate with either an *endo*- or *exo*-tet configuration, which is captured as a 'polyether epoxide hydrolase' annotation. Annotators can also return more than one possible reaction plan, in cases where more than one reaction is possible: for instance, the presence of a tryptophan dioxygenase alongside a nonribosomal peptide containing more than one tryptophan residue.

The function of the *reaction* is to actually execute the reaction as unambiguously specified by a single reaction plan. (If ambiguity is somehow introduced by accident during the course of prediction, the reaction will throw an exception). This is accomplished using a series of chemical operations

defined within PRISM as functions that provide wrappers to the Chemistry Development Kit. Most reaction classes further include *connectivity checks* to establish the chemical plausibility of a given reaction. For instance, if a particular hydroxyl group is slated to be phosphorylated, but that same hydroxyl has already been glycosylated during the course of reaction execution, the reaction class will throw an exception rather than generating an oxonium ion species. (For some tailoring reactions with extremely large combinatorial search spaces, a specific *reaction overlap check* is performed to exclude implausible combinations of reaction plans *a priori*, as described further below). The output of a reaction is a transformed chemical graph.

The final two aspects of a tailoring reaction are its *priority* and an indicator of whether it can act more than once. Reaction priority refers to the fact that biosynthetic pathways often require a specific sequence of reactions. For instance, a transamination reaction might first require oxidation of a hydroxyl group to a ketone. During library generation, reactions are sorted by their priority to act on the chemical graph in sequence. Reaction priorities within PRISM can take on arbitrary real values in order to flexibly account for sometimes conflicting requirements within and across biosynthetic families. Finally, it is conceivable that a given BGC could contain two copies of a given enzyme, but it would be meaningless for the reaction to proceed twice. For instance, tryptophan dimerization, as catalyzed by the chromopyrrolic acid synthase RebD, can only occur once between any pair of tryptophan residues. Thus, if a particular BGC should happen to contain two copies of RebD enzymes, only one is assumed to be active. This provides an additional layer of robustness to unexpected biosynthetic enzyme content within undiscovered BGCs.

The process of tailoring reaction enumeration involves the generation of a list of possible reaction plans for each tailoring enzyme by the annotators associated with that reaction. The reactions themselves are carried out later, during the process of combinatorial library generation (described further below).

Combinatorial plans. The process of tailoring reaction enumeration involves the generation of all possible reaction plans, up to some limit, by annotators for each tailoring enzyme. The output is thus a list of lists of reaction plans for each chemical graph generated in the preceding step. The next step in combinatorial structure prediction is to enumerate all possible combinations involving a single chemical graph and a single permutation of tailoring reactions, called a *combinatorial plan*. The execution of a single combinatorial plan leads to the generation of a single predicted structure within PRISM.

Combinatorial plan generation involves enumerating all combinations of possible tailoring reactions for each chemical graph. Repeating this process for all chemical graphs gives the total set of combinatorial plans to be explored during library generation. In cases where exhaustive enumeration of the entire combinatorial space is infeasible due to its size, a certain maximum number of combinatorial plans (set to 1,000 by default) is sampled, with combinations of reaction plans sampled equally from each chemical graph. Although some families of secondary metabolites do indeed have very large combinatorial search spaces (e.g., aminoglycosides or thiopeptides), we emphasize here that most do not: the median number of structures predicted for each BGC of the 'gold standard' set is four (Supplementary Data 2). The total number of predicted structures retained for each BGC is reduced in part by maximizing the number of tailoring reactions that could successfully be carried out, as described further below.

Library generation. Having generated a set of combinatorial plans, PRISM next seeks to execute each combinatorial plan to generate a complete predicted chemical structure. This process involves making

a deep copy of the information contained within the chemical graph of that combinatorial plan, and then executing each reaction in order of its priority on the copied graph in sequence. Both the chemical graph and each intermediate in the sequence of reactions are retained, and output in JSON format as the complete biosynthetic pathway for that predicted molecule.

As a sequence of reactions is carried out, some may fail the ‘connectivity checks’ described above. PRISM keeps track of the total number of reactions that could be successfully executed for each combinatorial plan. Furthermore, PRISM calculates the total number of disconnected subgraphs in the final product of each predicted structure. Only the set of predicted structures that simultaneously maximize the number of successful tailoring reactions, and minimize the number of disconnected subgraphs, are retained in the final library of predicted structures. These criteria fit the general intuition that we should use as much biosynthetic information as we have access to in structure prediction, while minimizing the amount of chemical matter that cannot be inferred to participate in any reaction at all. However, if it is not possible to connect all subgraphs within a single structure (for example, a sugar is activated, but no glycosylation site can be identified within the molecule), a molecule containing more than one disconnected subgraphs will be output to the user. In cases where many possible structures optimize both criteria equally well, PRISM imposes a maximum on the total number of structures output to the user; this is set by default to 50, but can be increased to any point up to 1,000. Predicted structures are output in SMILES format, and can be visualized using an interactive structure browser in the PRISM web application.

Hexose and deoxy sugar prediction

Secondary metabolites of many different biosynthetic families contain sugar moieties derived from primary and/or specialized metabolism. It was therefore necessary to develop a single system for sugar prediction within PRISM, agnostic to the particular biosynthetic family of a BGC of interest. However, despite the vast amount of accumulated knowledge about their enzymology and biosynthesis[27], a number of key challenges complicate the automated prediction of the sugar moieties in secondary metabolites. First, secondary metabolites often contain multiple sugars, including combinations of hexose and deoxy sugars. Second, for metabolites with multiple deoxy sugar moieties, individual enzymes may be shared between multiple sugar biosynthesis pathways, and enzymes from each pathway may not be physically separated within a given BGC. We previously described an algorithm that simultaneously infers the number and identities of hexose and deoxy sugars[10,11]; this algorithm subsequently interfaces with the PRISM structure prediction engine in order to enumerate all possible patterns of glycosylation of the core scaffold. The first step is to infer the numbers of hexose and deoxy sugars, respectively, on the basis of the number of glycosyltransferase enzymes within the BGC of interest. The number of glycosyltransferases is an imperfect marker for the number of sugar moieties in the final product, since some can act iteratively whereas others may be inactive, but we nevertheless found this heuristic to produce reasonable predictions in the vast majority of cases.

Identified glycosyltransferases are subsequently analyzed using a BLAST database of annotated hexose and deoxy sugar glycosyltransferases, and if the top-scoring hit is to a hexose glycosyltransferase, the substrate of the enzyme (one of glucose, mannose, gulose, or N-acetylglucosamine) is inferred to be that of the annotated glycosyltransferase. This approach relies on the observation that hexose-activating glycosyltransferases form a small number of distinct clades in a phylogenetic analysis of secondary metabolite glycosyltransferases. The limitations of homology-based inference of hexose sugar identity are further mitigated by the fact that three of the

four hexose sugar moieties are distinguished only by their stereochemistry, which is not incorporated within PRISM.

The remaining glycosyltransferases are assumed to transfer deoxy sugars. To predict the identifies of these sugars, as well as their number, we sought to compile a comprehensive database enumerating the complete biosynthetic pathways of secondary metabolite deoxy sugars. We revised and substantially expanded the glycogenetic code described by Kersten et al.[28], developing HMMs for 26 enzymes involved in deoxy sugar biosynthesis (e.g., 4,6-dehydratase or 3-ketoreductase), and recording the enzymatic functionalities required for the biosynthesis of 64 known deoxy sugars.

The identities of the deoxy sugars produced by a given BGC are then inferred using a strategy which seeks to maximize the overlap between known and inferred pathways. Briefly, for a number of deoxy sugar glycosyltransferases *n*, all possible combinations of deoxy sugars of size exactly *n* are enumerated (for $n \geq 4$, where the search space is >750,000 combinations, a random sample of 1,000 combinations is retained). Each possible combination is considered, with the aim of simultaneously minimizing both the number of enzymes in the known deoxy sugar biosynthetic pathways that were not detected in the BGC, and the number of enzymes in the BGC that are not incorporated within the known pathways. If more than 100 possible combinations minimizes both objectives, only the first 100 are retained. Finally, the identified hexose sugar(s) are added to each combination.

Having thereby predicted the combination(s) of hexose and/or deoxy sugars decorating an encoded secondary metabolite, all possible glycosylation patterns are enumerated within the PRISM structure prediction engine. Glycosylation is assumed to occur at any possible oxygen or indole nitrogen within the chemical graph. An independent HMM was also constructed for a distinct clade of type II polyketide C-glycosyltransferases[29]; these are assumed to catalyze sugar transfer at the α-carbon of any type II polyketide residue. Glycosylation reactions for each sugar within a combination are executed independently, enabling prediction of oligosaccharide chains in addition to glycosylation patterns in which multiple sugars are transferred to distant sites on the aglycone.

New biosynthetic classes in PRISM 4

To achieve complete coverage of biosynthetic families of bacterial secondary metabolite antibiotics that are currently in clinical use, we developed chemical structure prediction functionality for seven new classes of secondary metabolites (alkaloids, aminoglycosides, β-lactams, lincosamides, nucleosides, phenazines, isopropylstilbenes), and expanded chemical structure prediction for RiPPs, nonribosomal peptides, and type I polyketides. Here, we provide a comprehensive description of the new hidden Markov models and virtual tailoring reactions developed within PRISM to account for these aspects of secondary metabolite biosynthesis.

Expansion of thiotemplated chemical structure prediction

We took a systematic approach to expand the scope of chemical structure prediction from canonical thiotemplated (i.e., nonribosomal peptide or type I polyketide) pathways. First, we identified specific conserved chemotypes that were poorly predicted within PRISM. Second, we identified rare monomers that were not predicted within PRISM.

New thiotemplated chemotypes in PRISM 4. We developed functionality to accurately predict complete chemical structures from secondary metabolic chemotypes that were poorly predicted in previous versions of PRISM, including tetrahydroisoquinolines, pyrrolobenzodiazepines, polyketide macrolactams, polyether antibiotics, lipocyclocarbamates, pyrrolizidine alkaloids, tetramic acid polyketides, chloramphenicol, albicidin, and cyclomarin.

Tetrahydroisoquinolines. We developed a suite of 19 HMMs, and 12 virtual tailoring reactions, to predict the chemical structures of tetrahydroisoquinolines, a complex family of extensively tailored nonribosomal peptides[30], of which ET-743 (Yondelis) is a clinically used anticancer agent. The core of these molecules is assembled by an iterative, multi-step reaction catalyzed by the unique Pictet-Spengler condensation domain, which results in assembly of the core from two modified tyrosine residues and the product of the remainder of the nonribosomal peptide synthetase machinery. In biosynthesis of tetrahydroisoquinoline pyrrolidine metabolites, including quinocarcin and napththyridinomycin, the second modified tyrosine is replaced by an arginine residue.

We first developed new substrate-specific adenylation domain models for the nonproteinogenic amino acids activated by tetrahydroisoquinoline nonribosomal synthetases, including two distinct clades of 3-hydroxy-5-methyl-O-methyltyrosine adenylation domains and glycolic acid-activating adenylation domains involved in tetrahydroisoquinoline pyrrolidine biosynthesis. These modified tyrosine adenylation domains require the presence of the tyrosine 3-C-methyltransferase SfmM2 and the 3-methyltyrosine peroxygenase SfmD for substrate prediction. The glycolic acid adenylation domain requires the presence of the α and β D-xylulose 5-phosphate dehydrogenase subunits Qcn7 and Qcn9. Next, we developed HMMs for two phylogenetically distinct clades of reductase domains found in tetrahydroisoquinoline biosynthesis (SfmC-type and Qcn17-type). The identification of the Pictet-Spengler condensation domain and a tetrahydroisoquinoline reductase provides the basis for the formation of the tetrahydroisoquinoline core within PRISM, conditioned on the adenylation domain substrates identified. If the cluster contains at least one modified tyrosine adenylation domain and no arginine adenylation domain, the Pictet-Spengler domain is assumed to activate two modified tyrosine residues; if an arginine-activating adenylation domain is present, one is replaced by arginine. Conversely, if the cluster contains phenylalanine- and arginine-activating adenylation domains, the Pictet-Spengler domain is assumed to activate phenylalanine and arginine. The Pictet-Spengler domain then catalyzes the formation of the core of the metabolite, the latter involving a spontaneous Mannich reaction.

Next, we developed HMMs and virtual tailoring reactions for the numerous enzymes that tailor this core to produce the final products. The tetrahydroisoquinoline N-methyltransferase SfmM1 is assumed preferentially to act at the backbone nitrogen of a modified tyrosine residue, but is permitted to act at any free nitrogen if no such atom exists. The quinone hydroxylases SfmO2 and SfmO4 each catalyze quinone formation at a modified tyrosine residue. The SfmE peptidase catalyzes cleavage of the acylpeptidyl nonribosomal peptide synthetase product; within PRISM, it is assumed to have a relaxed substrate specificity, potentially cleaving any amide bond. The SfmCy2 transaminase installs an an α-keto group at the new nitrogen terminus introduced by the SfmE peptidase. In tetrahydroisoquinoline pyrrolidine pathways, a similar reaction is catalyzed by the Qcn1 esterase, which preferentially acts at the glycolic acid moiety, but is assumed to potentially hydrolyze any ester or amide bond if no such moiety is found. The Qcn18 dioxygenase catalyzes the formation of dehydroarginine from arginine, a transformation required for pyrrolidine ring formation. Similarly, the Qcn4 phenylalanine *meta*-hydroxylase catalyzes the formation of the quinocarcin 'modified tyrosine'-like residue from phenylalanine. The split dehydrogenase subunit proteins Qcn2 and Qcn3 catalyze oxidation of a terminal aldehyde to a carboxylic acid; both subunits are required for the reaction to proceed. The Qcn5 O-methyltransferase is permitted to act at any free hydroxyl. In napthyridinomycin-type pathways, the Cya28 enzyme is assumed to mediate aminoacetal formation. Finally, we built HMMs for the SfmM3 -methyl-4,5-dihydroxyphenylalanine 4-O-methyltransferase and

the Qcn10 ACP-glycolyl ketosynthase, but did not associate these with either an adenylation domain substrate prerequisite or a tailoring reaction.

Pyrrolobenzodiazepines. Pyrrolobenzodiazepines are a family of antineoplastic dipeptides produced by dimodular nonribosomal synthetases and whose core comprises an anthranilic acid and proline diresidue, both of which may be further modified. The majority of pyrrolobenzodiazepines contain heavily modified proline residues derived from tyrosine, using enzymatic machinery shared with metabolites of the lincomycin family; the biosynthesis of these residues is discussed below. However, we also developed a number of HMMs designed to predict the anthranilic acid-derived residue. In BGCs that contain homologs of the phenazine anthranilate synthase PhzE and isochorismatase PhzD, the resulting *trans*-2,3-dihydro-3-hydroxyanthranilic acid residue may be oxidized by the Lim4 oxidoreductase to produce 3-hydroxyanthranilic acid, which may be incorporated directly by the nonribosomal peptide synthetase or be further tailored by the dual-component 4-monooxygenase Lim7/Lim8 or the O-methyltransferases Lim9 or Por26 (permitted to act at any hydroxyl group). Alternatively, the anthranilic acid residue may be derived from kynurenine, via the sequential action of the SibC kynurenine 3-monooxygenase and the SibQ kynureninase. Kynurenine-derived anthranilic acid residues may also be 4-methylated by the SibL kynurenine C-methyltransferase, or 5-hydroxylated by the SibG monooxygenase. Finally, in tomaymycin-family pathways, the anthranilic acid residue can be provided by an anthranilate synthase (TomP), and then derivatized by the dual-component 4-monooxygenase TomE/TomF (homologous to Lim7/Lim8), the 5-monooxygenase TomO, and the O-methyltransferase TomG (permitted to act at any hydroxyl group). We additionally developed new substrate-specific adenylation domain models for the anthranilate and proline derivatives incorporated by pyrrolobenzodiazepine nonribosomal peptide synthetases. Finally, we developed a model for the phylogenetically distinct clade of nonribosomal peptide synthetase reductase domains involved in pyrrolobenzodiazepine biosynthesis, which are assumed to catalyze either linear aldehyde or cyclic imine formation to assemble the final product.

Polyketide macrolactams. To predict the chemical structures of the large family of β-amino acid-containing macrolactam polyketides, such as vicenistatin, incednine, salinilactam, hitachimycin, or cremimycin among many others[22], we first developed several new substrate-specific adenylation domain models for nonproteinogenic amino acids characteristically found in specific macrolactam subfamilies, including methyl-aspartic acid (vicenistatin, ciromicin), 3-aminobutyric acid (incednine, salinilactam, micromonlactam, lobosamide A, mirilactam A), and β-phenylalanine (hitachimycin). We additionally built HMMs for domains involved in the biosynthesis of these monomers, including the methylaspartate mutase σ and ε subunits VinH and VinI; the glutamate 2,3-aminomutase and β-glutamate decarboxylase IdnL4 and IdnL3; and the phenylalanine 2,3-aminomutase HitA. Prediction of 3-aminobutyric acid requires IdnL3 and IdnL4, whereas prediction of β-phenylalanine requires the presence of HitA. These adenylation domains are often found as standalone proteins, which necessitated revising the rules for subgraph generation, as standalone adenylation domain proteins are assumed by default not to activate a residue for biosynthesis (they are assumed instead to either be inactive, or involved in precursor biosynthesis of rare monomers). We additionally developed HMMs for the VinK and HitC acyltransferases and the VinJ peptidase, which are involved in installation and removal of a terminal alanyl protecting group moiety on the nitrogen terminus of biosynthetic intermediates; however, for simplicity, PRISM does not actually include the protection–deprotection sequence within the *in silico* biosynthetic pathway.

Next, we designed several tailoring enzyme HMMs and virtual tailoring reactions to account for remaining features of macrolactam polyketide biosynthesis. In particular, we created an HMM for the PLP-dependent iso-aspartate decarboxylase VinO, which converts methyl-aspartic acid to 3-aminoisobutyrate in vicenistatin-type macrolactams. For macrolactams containing a 3-amino fatty acid, such as cremimycin, heronamide A, BE-14106, or ML-449, we also developed models for the putative thioesterase homolog CmiS1, which is assumed to catalyze Michael addition of a glycine residue to the a polyketide backbone double bond, and the FAD-dependent glycine oxidase CmiS2, installing a backbone nitrogen in the polyketide chain and releasing glyoxylate[31]. Finally, we constructed HMMs and virtual tailoring reactions for the six genes (HitM1–M6) involved in biosynthesis of the five-membered carbocycle (and its O-methylated variant) found in hitachimycin and cremimycin-type pathways[32]. The HitM1–M5 reactions occur at a sequence of three ketide residues, where the first residue is reduced to a hydroxyl and the second and third residues are dehydrated. The HitM1 dehydrogenase/reductase is assumed to oxidize the first residue, restoring the ketone, whereafter the HitM3 monooxygenase installs an α-keto group. Either of the isomerase-type enzymes HitM2 or HitM5 is then assumed to be sufficient to catalyze cyclization to form a substituted cyclopentenone moiety, which is reduced by the HitM4 dehydrogenase/reductase and optionally O-methylated by HitM6, which is permitted to act at any free hydroxyl.

Polyether antibiotics. Polyether antibiotics are structurally complex type I polyketides, widely used in veterinary medicine, containing two or more cyclic ether or acetal rings that are installed by a cascade of nucleophilic attacks involving the polyketide backbone. We developed HMMs and virtual tailoring reactions to account for the complex course of polyether biosynthesis, including the polyether epoxidase and the split epoxide hydrolase proteins (MonBI- and MonBII-type) that initiate the cascade of cyclizations. By default, the polyether epoxidase is assumed to epoxidize the most carboxy-terminal ketide double bond in the thiotemplated subgraph. However, if the BGC does not contain either of the split epoxide hydrolase proteins, the polyether epoxidase is treated as a generic polyketide epoxidase, and permitted to act at any backbone double bond. The epoxide hydrolase is found either as a multi-domain protein or as split proteins in polyether BGCs; the presence of either HMM is sufficient to catalyze the epoxide-opening cascade of nucleophilic attacks, which is perhaps the most complex reaction within PRISM. Beginning at the epoxide group, the annotator first proceeds away from the carboxy terminus of the thiotemplated subgraph of the molecule in steps of two residues. At each residue, the annotator makes a decision whether to continue extending the reaction plan or terminate the chain of nucleophilic attacks. If the residue in question is a ketone, the annotator proceeds to take another two-residue step away from the carboxy terminus. If the residue in question is a double bond, the annotator records epoxidation of the double bond, and takes another step. If the residue in question is a alcohol, the annotator assumes that the cascade of nucleophilic attacks starts at that residue. (If the annotator encounters a single bond, an error is thrown). Finally, the annotator also attempts to step towards the carboxy terminus, asking whether the nucleophilic attack cascade can continue downstream of the most carboxy-terminal epoxide group at one or more unreduced ketide residues. The reaction itself first installs epoxide groups at double-bonds inferred to be part of the nucleophilic attack cascade, then carries out the sequence of nucleophilic attacks itself. If the carboxy-terminal residue is an epoxide group, either *endo*- or *exo*-tet cyclization is permitted[33]. This system mechanistically accounts for the biosynthesis of the known polyether antibiotics, including monensin, salinomycin, lasalocid, and nanchangmycin among others, with the exception of cyclic moieties that are installed by distinct enzymes, such as the first pyran ring in salinomycin biosynthesis[34].

Lipocyclocarbamates and pyrrolizidine alkaloids. To account for the biosynthesis of these related families of modified nonribosomal peptides, we developed HMMs and virtual tailoring reactions for the LpiC[35] and PxaB[36] monooxygenase families. Both enzymes act at a carboxy-terminal proline-serine diresidue. The proposed biosynthetic pathway involves serine dehydration by an unusual catalytic thioesterase domain, followed by spontaneous cyclization to a tetrahydroindolizine dione; however, because of the relatively weak correlation between thioesterase phylogeny and specificity[37], we opted to associate this reaction with the phylogenetically distinct monooxygenases, with the goal of increasing both sensitivity and specificity. From this common intermediate, LpiC and PxaB are then each assumed to catalyze Baeyer–Villiger oxidation, albeit with different courses of ring rearrangement and decarboxylation leading to the formation of the lipocyclocarbamade or pyrrolizidine cores, respectively.

Tetramic acid polyketides. To account for the biosynthesis of tetramic acid-containing or pyridone-based type I polyketides, such as tirandamycin B, streptolydigin, α-lipomycin, or kirromycin, we developed a HMM and tailoring reaction for the TrdC Dieckmann cyclase[38]. The reaction is assumed to occur at a carboxy-terminal malonate–amino acid diresidue, with Dieckmann cyclization leading to tetramate formation (or, when the carboxy-terminal amino acid is a β-amino acid, formation of a piperidine dione that is subsequently tailored to yield the pyridone moiety).

Chloramphenicol. Choramphenicol is a structurally unique broad-spectrum antibiotic whose biosynthesis involves nonribosomal peptide synthetase machinery, but which eluded the rule-based BGC detection system implemented in the original PRISM release. To identify chloramphenicol-family BGCs, we designed a new rule for thiotemplated BGC identification, requiring the presence of the 4-amino-4-deoxyprephenate dehydrogenase CmlC and the dichloroacetate transferase CmlG. Chemical structure prediction was achieved by creating HMMs and virtual tailoring reactions for the chorismate transaminase CmlB, which activates a chorismate residue and produces 4-amino-4-deoxychorismate; the chorismate mutase CmlD, which converts this to 4-amino-4-deoxyprephenate; and the cyclohexadienyl dehydrogenase CmlC, which is assumed to produce *para*-aminophenylalanine. This is followed by β-hydroxylation by CmlA and N-oxygenation by CmlI to produce *para*-nitrophenylserine. In parallel, the acetyl-ACP halogenase CmlS activates and dichlorinates the α-carbon of an acetyl group, and CmlH catalyzes amide bond formation with the backbone nitrogen of the *para*-nitrophenylserine residue. Finally, the phylogenetically distinct nonribosomal peptide reductase and the aldehyde reductase CmlJ catalyze sequential reduction of the *para*-nitrophenylserine carboxylic acid to a primary alcohol.

Albicidin. Albicidin and the structurally related product cystobactamid are structurally unique natural products characterized by multiple aromatic δ-amino acids, among other nonproteinogenic amino acids. We built a number of different substrate-specific adenylation domain models, tailoring domain HMMs, and virtual tailoring reactions to predict the chemical structures for metabolites of this family[39]. First, we built new substrate-specific adenylation domain models for several nonproteinogenic amino acids present in albicidin/cystobactamid, including *para*-hydroxybenzoic acid, *para*-aminobenzoic acid, and *para*-aminohydroxybenzoic acid. We also developed HMMs for tailoring domains involved in the biosynthesis of these nonproteinogenic amino acids, including the chorismate-pyruvate lyase Alb20, the *para*-hydroxybenzoate-CoA ligase Alb07, and the *para*-aminobenzoate *meta*-hydroxylase Alb20. Prediction of *para*-hydroxybenzoic acid requires the presence of Alb07 and Alb20; prediction of

para-aminobenzoic acid requires the presence of the CmlB chorismate transaminase, described above; prediction of *para*-aminohydroxybenzoic acid requires CmlB and Alb12. Of note, these models and prerequisites are distinct from PRISM's generic models for *para*-hydroxybenzoic acid and *para*-aminobenzoic acid activation, due to the distinct primary sequences of the domains involved in albicidin biosynthesis[40]; the generic models to not require any additional tailoring domains as prerequisites. In addition, we built HMMs and virtual tailoring reactions for the Alb02 O-methyltransferase (permitted to act at any free hydroxyl), the Alb08 and CysP *ortho*-hydroxylases (permitted to act at any phenyl ortho carbon), and the CysS C-methyltransferase, which catalyzes iterative dimethylation of a single methyl group. The cyanoalanine biosynthesis domain of Alb04 is assumed to catalyze the conversion of an asparagine residue into cyanoalanine. Finally, we revised the system for installation of amide linkages between adjacent residues to allow backbone δ-amino acid amide bond formation between aromatic amino acids.

Cyclomarin. Cyclomarins are cyclic nonribosomal peptides that contain a number of nonproteinogenic amino acid residues, several of which are present in related products such as ilamycins and rufomycins[41]. We developed a number of new substrate-specific adenylation domain HMMs, tailoring domain HMMs, and virtual tailoring reactions to account for the biosynthesis of these complex nonribosomal peptides. These include the tryptophan N-isoprenyltransferase CymD, which catalyzes isoprenylation of an indole nitrogen; the cytochrome P450 CymV, which catalyzes epoxidation of a prenyl group; the cytochrome P450 CymO, which catalyzes β-hydroxylation of a phenylalanine residue; the cytochrome P450 CymS, which catalyzes δ-hydroxylation of a leucine residue; the O-methyltransferase CymP, which is permitted to act at any free hydroxyl; the α-ketoglutarate-dependent dioxygenase CymW, which catalyzes β-hydroxylation of a tryptophan residue; the cytochrome P450 RufO, which catalyzes *meta*-nitration of any phenyl group; the cytochrome P450 IlaD, which catalyzes α-oxidation to install a α-keto group at any ketide residue; and the transaminase IlaH, which catalyzes transamination of the α-keto group. We additionally built a substrate-specific adenylation domain HMM for the unusual amino acid 2-amino-3,5-dimethylhex-4-enoic acid, which is derived from the condensation of isobutyraldehyde and pyruvate. Substrate prediction requires the presence of the complete biosynthetic pathway to this residue, including the isobutyryl-CoA dehydrogenase CymE, the isobutyrylaldehyde aldolase CymF, the 2-keto-4-hydroxy-isoheptanate hydratase CymH, and the SAM-dependent C-methyltransferase CymB.

Unusual monomer biosynthesis in PRISM 4. We next developed a series of substrate-specific models, complemented by series of virtual tailoring reactions and prerequisite domains for rare monomer biosynthesis, in order to predict more than a dozen new unusual monomers, in addition to those described above.

Piperazic acid. To predict the presence of piperazic acid in nonribosomal peptides, we revised and expanded the existing substrate-specific adenylation domain model for piperazic acid in PRISM, and developed a model for the heme-dependent nitrogen–nitrogen bond-forming enzyme KtzT, which is required for substrate prediction[42].

para-Aminobenzoic acid. To predict *para*-aminobenzoic acid moieties, found in secondary metabolites from a number of different biosynthetic families, we built HMMs for the aminodeoxychorismate synthase PabB and the aminodeoxychorismate lyase PabC. PabB is assumed to catalyze a

chorismate residue to catalyze aminodeoxychorismate synthesis, with PabC thereafter catalyzing its conversion to *para*-aminobenzoic acid.

Homotyrosine. To predict homotyrosine residues, we built HMMs for the benzylmalate synthase HphA and the benzylmalate isomerases HphB, HphC, and HphD, as well as a new homotyrosine adenylation domain model; however, since the HphBCD genes may be found in *trans* to a homotyrosine-containing BGC, these are not prerequisites for substrate prediction.

Enduracididine. To predict enduracididine residues, we developed two substrate-specific HMMs for distinct clades of enduracididine-activating adenylation domains. We additionally developed HMMs for a series of tailoring domains involved in enduracididine biosynthesis, including the arginine γ-monooxygenase/transaminase MppP, the α-keto-enduracididine cyclase MppR, and the enduracididine transaminase MppQ. Prediction of enduracididine as an adenylation domain substrate requires the presence of all three in the BGC. Finally, we developed a HMM and virtual tailoring reaction for the enduracididine β-hydroxylase MppO.

4-methylproline. To predict the presence of 4-methylproline, as found in a number of cyanobacterial pathways[43], we developed HMMs for the methylpyrroline cyclase NosE and the methylpyrroline reductase NosF, as well as a 4-methylproline substrate-specific adenylation domain. The presence of both NosE and NosF is required in order to permit prediction of 4-methylproline.

Tambroline. To predict the presence of the lysine-derived nonproteinogenic amino acid tambroline[44], we developed a substrate-specific adenylation domain model, as well as models for the TbrP lysine dehydrogenase and TbrQ cyclase involved in its biosynthesis, which are required for substrate prediction.

Arizidine-containing amino acids. We developed a suite of HMMs and tailoring reactions to account for the biosynthesis of arizidine-containing amino acids, as found in metabolites such as vazabitide[45] or ficellomycin[46]. We first built HMMs for the series of tailoring enzymes involved in the biosynthesis of the nonproteinogenic amino acid (2S,6R)-diamino-(5R,7)-dihydroxy-heptanoic acid (DADH) from glutamate. These include a series of enzymes with homologs involved in lysine biosynthesis, including the LysW homolog amino-group carrier protein Vzb22; the LysX isopeptide bond-forming ligase homolog Vzb23; the LysZ glutamate kinase homolog Vzb25; the LysY glutamyl-phosphate reductase homolog Vzb24; and the LysK peptidase homolog Vzb26. We additionally built HMMs for the transketolase N- and C-terminal domain proteins Vzb27 and Vzb28, which are specific to DADH biosynthesis; the transaminase Vzb9; and a substrate-specific adenylation domain model. Substrate prediction of DADH requires the presence of Vzb27 and Vzb28. We next designed virtual tailoring reactions for the lysine dehydrogenase TbrP and cyclase TbrQ, described above in the context of tambroline biosynthesis, and whose homologs are involved in biosynthesis of a similar five-membered nitrogen-containing ring in arizidine-containing metabolites. These domains are therefore used both as prerequisites to permit substrate prediction of the rare nonproteinogenic amino acid tambroline, and alternately as tailoring enzymes with activity specific to DADH residues, with TbrP catalyzing α,β-dehydrogenation and TbrQ catalyzing cyclization of the pyrrolidine ring. The Fic28 (Vzb21) sulfotransferase then catalyzes arizidine formation, yielding the bicyclic amino acid found in vazabitide. Finally, to account for the presence of the guanidino group in the ficellomycin bicyclic monomer, we built HMMs and tailoring reactions for the dehydrogenase and transaminase pair Fic13

and Fic16 and the amidinotransferase Fic36, which are assumed to sequentially convert the 4-hydroxyl group to a ketone and then a primary amine, and install the guanidino group, respectively.

Dihydroxyhexane carboxylic acid. To predict dihydroxycyclohexane carboxylic acid biosynthesis from shikimate, as found in the enacyloxin biosynthetic pathway[47], we developed HMMs for shikimate dehydratase (Bamb_5916), two shikimate reductases (Bamb_5914/Bamb_5918), shikimate isomerase (Bamb_5912). Within PRISM, Bamb_5916 is assumed to activate a shikimate residue, and Bamb_5914 and Bamb_5918 are assumed to catalyze 1,2 and 5,6 double bond reduction, respectively, with the remainder of the pathway implemented according to Mahenthiralingam et al.[47]. Finally, the DHCCA condensation enzyme Bamb_5915 is assumed to catalyze ester bond formation between the C3 hydroxyl group of the shikimate-derived residue and any free carboxylic acid.

3-Amino-6-hydroxy-2-piperidone. To predict the formation of this moiety from glutamate, as is found in a number of cyanobacterial peptides of the cyanopeptolin family, we developed a HMM and virtual tailoring reaction for the 3-amino-6-hydroxy-2-piperidone cyclase ApdF, which is permitted to act at any glutamate residue within a molecule. We also developed a new substrate-specific HMM for cyanobacterial glutamate-activating adenylation domains, which we observed to be poorly predicted by existing models.

2-Carboxy-6-hydroxyoctahydroindole. To predict the presence of this unusual moiety, found in a number of cyanobacterial nonribosomal peptides[48], we developed HMMs and virtual tailoring reactions for the AerC prephenate transaminase, which activates and oxygenates an arogenate residue; the cupin domain-containing protein AerE, which catalyzes Michael addition to form a bicyclic intermediate; and the AerD decarboxylase and AerF reductase, which catalyze the last steps in 2-carboxy-6-hydroxyoctahydroindole formation. In addition, we developed a substrate-specific model for the 2-carboxy-6-hydroxyoctahydroindole adenylation domain, requiring the presence of AerD, AerE, and AerF in the BGC for substrate prediction.

6-Chloro-4-hydroxyindole-3-carboxylic acid and α-methylserine. These residues are found in several known metabolites, including JBIR-34 and tambromycin[44,49]. We developed a substrate-specific adenylation domain model, as well as HMMs for tailoring enzymes implicated in its biosynthesis, including the 4- and β-dihydroxylating cytochrome P450 monooxygenase FmoC, the aldehyde dehydrogenase FmoI, and the aldolase FmoM. All three of FmoC, FmoI, and FmoM are required to predict 6-chloro-4-hydroxyindole-3-carboxylic acid as an adenylation domain substrate. We also developed a substrate-specific adenylation domain model for α-methylserine, which requires the presence of the α-methylserine synthase AmiS to permit substrate prediction.

Expansion of RiPP chemical structure prediction

We extended our previously described system for genome-guided RiPP chemical structure prediction[6] in two ways. First, we developed BGC identification and structure prediction functionality for three new classes of RiPPs, including phosphoramidates, pantocins, and a recently described family of β-amino acid-containing peptides[50], by compiling eleven new HMMs and eight new reactions. Second, we refined existing RiPP virtual tailoring reactions with a focus on lowering the combinatorial search space when this was justified on the basis of known RiPP biosynthesis, and revising reactions for consistency with biochemical results published since the descripion of the original system.

To predict the chemical structures of phosphoramidate-containing RiPPs, we developed HMMs for the dual phosphoramidation/adenylation domain MccB, the efflux pump MccC, and MccD and the N-terminal domain of MccE, which together are required for aminopropylation of the phosphate group[51]. Detection of a putative phosphoramidate BGC requires the presence of both MccB and MccC homologs. Because the diversity of known precursor peptides precluded the construction of an accurate HMM for precursor detection, these are identified using a permissive heuristic: any ORF of length 180 nt or less ending in an asparagine residue within a phosphoramidate BGC is considered a potential precursor. MccB is assumed to catalyze the simultaneous conversion of the carboxy-terminal asparagine residue to an aspartate amide and addition of either adenosine or cytidine monophosphate to the carboxy-terminal nitrogen atom by a phosphoramidate bond, with the reaction proceeding via a succinimide intermediate. MccD and MccE are assumed to jointly catalyze the attachment of an aminopropyl group to the phosphate; the presence of both domains is required for this reaction to proceed. Finally, because some phosphoramidate-containing RiPPs are products of proteolytic cleavage by *trans*-acting proteases[52], all possible sites of proteolytic cleavage resulting in peptides of at least seven amino acids are enumerated. For reaction plans with no proteolytic cleavage (i.e., with the full-length ORF intact), the N-terminus of the precursor is formylated.

To predict the chemical structures of pantocins, we developed HMMs for the pantocin cyclase PaaA, the decarboxylase-cyclase PaaB, the efflux pump PaaC, and the precursor peptidase PaaD. The cyclase PaaA and the decarboxylase-cyclase PaaB are required to detect putative pantocin BGCs. The diversity of known precursor peptides from RiPPs of this family did not permit the construction of an accurate HMM for the precursor peptide, so these are identified using a permissive heuristic: any ORF of length 30–150 nt, containing an EE diresidue five or more amino acids from its C-terminus, within a pantocin BGC is considered a potential precursor. PaaA is assumed to catalyze the formation of the first ring at any glutamate–glutamate diresidue, with PaaB catalyzing Claisen condensation and oxidative decarboxylation[53]. PaaD is assumed to subsequently catalyze peptide bond cleavage to yield the processed tripeptide; however, as some pantocin-family BGCs apparently lack the peptidase, its activity is assumed to be supplied in *trans* when the PaaD model is not detected.

To identify and predict a recently identified of RiPPs containing β-amino acids, whose formation is mediated by tyramine excision and which are exemplified by the *plp* and *pcp* loci from Pleurocapsa spp. PCC 7319 and 7327 (ref. [50]), we developed HMMs for the radical SAM splicase PlpX, its PqqD-family partner PlpY, and the Nif11-type precursor peptides (PlpA). Only PlpX is required for BGC detection, and is also assumed to be sufficient to catalyze α-keto-β-amino amide formation at tyrosine–glycine diresidues in the precursor peptide.

In addition to these new families, we also made a number of changes to existing reactions. The reaction catalyzed by the thioviridamide protein TvaD was updated based on the finding that the originally reported N-terminal moiety in thioviridamide was introduced during acetone extraction thioamide biosynthesis protein TvaH[54]. Similarly, the thioviridamide biosynthesis protein is now assumed to catalyze variable numbers of thioamide bond formations (between one and six, or two less than the total number of residues in the peptide in the event that this is less than six), and thioamides do not need to be adjacent. The glycocin SunS glycosyltransferase, originally assumed to be an S-glycosyltransferase, is permitted to act at all combinations of one or two serine or cysteine residues, with permutations of either glucose or N-acetylglucosamine[55]. A review of known thiopeptides was also performed to reduce the combinatorial search space when possible. This led to a number of changes, including reduction in the size range of macrocycles produced by pyridine formation (from 8–13 residues to 9–12 residues), a maximum of three instead of four

cyclodehydrations at serine or threonine residues, and a requirement that serine residues C-terminal to the first serine involved in pyridine formation and N-terminal to the second serine involved in pyridine formation form oxazoles, when possible.

Aminoglycosides

Aminoglycosides are a clinically important class of bactericidal antibiotics whose structures comprise a core aminocyclitol moiety decorated by one or more sugars, including deoxy and amino sugars[56]. The essential challenge in aminoglycoside structure prediction therefore consists of predicting the identity of the aminocyclitol core, and the number, identity, and glycosidic bond pattern linking the sugar moieities to this core. We adapted and extended the hexose and deoxy sugar inference algorithm within PRISM[10,11], described above, for aminoglycoside prediction as follows. First, we catalogued the inferred biosynthetic pathways for 20 aminocyclitols or amino sugars (Supplementary Table 4), and constructed HMMs for 63 known enzymes involved in aminoglycoside biosynthesis (Supplementary Data 1). Second, we developed a generic rule for aminoglycoside BGC detection, defined by the presence of either an inositol phosphate monophosphatase, the KasD 4,6 dehydratase, or a 2DOI synthase, and at least two other aminoglycoside biosynthesis genes. We additionally developed more stringent rules for the detection of sixteen specific aminoglycoside subtypes (Supplementary Table 3), in order to provide users with more information about specific chemotypes when this might be possible. Third, we developed an approach to simultaneously infer the number and identity of amino sugars. Whereas a reasonable estimate of the number of deoxy and hexose sugars in a metabolite is provided by the number of glycosyltransferases in the BGC, no such heuristic was immediately obvious for aminoglycosides. Thus, the method implemented within PRISM 4 begins by enumerating all possible combinations with repetitions of deoxy sugars of size one to four; the maximum of four sugars was chosen on the basis of the maximum number of sugar residues within any known aminoglycoside. For each sugar combination, the number of aminoglycoside genes within the BGC, but not any of the cassettes for the sugar combination, is calculated, as is the number of aminoglycoside genes within the cassettes for the sugar combination not found within the BGC. Combinations of sugars that minimize both numbers are retained as predictions for the aminoglycoside BGC. Finally, since glycosidic bonds cannot be predicted *a priori* from BGC sequence information alone, all possible patterns of glycosidic bonds is enumerated for each combination in order to generate a library of predicted structures. This leads to the largest combinatorial search space for any family of secondary metabolites (Supplementary Fig. 6); however, the resulting library of predicted structures exhibits a high degree of structural similarity, as members are distinguished primarily by the positions of the glycosidic bonds.

We validated the performance of our approach to aminoglycoside sugar prediction by comparison to a naïve approach, requiring an exact match between the aminoglycoside genes observed in a BGC of interest and the database of known aminoglycoside sugar cassettes. This method first retrieves all possible sugars that the observed BGC can produce, based on the requirement of an exact match between the biosynthetic gene cassette for that sugar and the aminoglycoside biosynthesis genes within this BGC. Subsequently, the method calculates all possible subsets of aminoglycoside sugars, and prioritizes those subsets with the smallest number of aminoglycoside biosynthesis genes in the BGC unaccounted for by biosynthesis of the sugar subset. Accuracy was calculated as the number of sugars correctly predicted divided by the total number of sugars in the predicted combination. When more than one combination was predicted for a BGC, the mean accuracy of all combinations was calculated. Relative to the exact match algorithm, the PRISM 4 method yielded significantly higher accuracy (mean accuracy of 76% vs. 46%; $p < 10^{-15}$, paired

Brunner–Munzel test; Supplementary Fig. 11a), and predicted a total number of sugars that was significantly closer to the true number (mean error of +0.02 sugars vs. –1.07 sugars; $p < 10^{-15}$, paired Brunner–Munzel test; Supplementary Fig. 11b).

β-lactams

We developed libraries of 40 HMMs and 28 virtual tailoring reactions to predict complete chemical structures for 11 classes of β-lactams with known biosynthetic pathways.

Clavams. Chemical structure prediction for β-lactams of the clavam family begins with the carboxyethylarginine synthase (CEAS)[57], which catalyzes the formation of N^2-(2-carboxyethyl)arginine from arginine and glyceraldehyde 3-phosphate. This is followed by β-lactam formation by the ATP/Mg^{2+}-dependent β-lactam synthetase (BLS)[58], and hydrolysis of the guanidino group by the proclavaminate amidino hydrolase (PAH), producing proclavaminic acid. The clavaminic acid pathway proceeds with the multi-step reaction catalyzed by the clavaminic acid synthase (CAS)[59], or its variant in clavulanic acid pathways, N-glycyl-clavaminic acid synthetase (GCAS). The clavaldehyde dehydrogenase (CAD) terminates clavulanic acid biosynthesis. Detection of putative clavaminic acid BGCs requires both of CEAS and BLS; clavulanic acid BGCs additionally require GCAS; and 5*S*-clavams additionally require the presence of the Cvm1 aldo-keto reductase [60].

Carbapenems. The carbapenem biosynthetic pathway begins with activation and dehydrogenation of a proline residue by the proline dehydrogenase CarD. Condensation with a malonate residue, activated and catalyzed by the crotonase-superfamily carboxymethylproline synthase CarB, produces carboxymethylproline, which is subsequently cyclized by the carbapenem β-lactam synthase CarA to produce carbapenem-3-carboxylate[61]. Finally, desaturation by the carbapenem synthase CarC yields the carbapenem. Alternatively, in complex carbapenems, such as theinamycin or PS-7, the carbapenem-3-carboxylate intermediate can be further derivatized by the radical SAM enzyme ThnK, which catalyzes two successive methylation reactions[62] to install a 6-ethyl group; 2,3-oxidation by the CarC homolog ThnG; and hydroxylation of the 6-ethyl group at the methylene position by the oxygenase ThnQ. As the enzyme responsible for pantetheine addition to the C-2 position is not known, all three of pantetheine residue activation, the addition reaction, and subsequent hydrolysis of pantothenic acid are associated with the pantetheine hydrolase ThnT. Finally, the N-acetyltransferase ThnF is permitted to act at any free nitrogen atom. We additionally built HMMs, but not virtual tailoring reactions, for the CarE 2Fe–2S ferredoxin; the ThnA oxidoreductase; and the putative split carboxylate reductase proteins ThnN and ThnO. Detection of putative simple carbapenem BGCs requires the presence of CarA, CarB, and CarC, whereas complex carbapenems require CarA, CarB, and ThnT.

Monobactams. To predict the thioesterase-mediated mechanism of monobactam formation that characterizes sulfazecin biosynthesis[63], we developed a HMM and virtual tailoring reactions for the specialized thioesterase domain (referred to as Monobactam_TE within PRISM), which catalyzes cyclization of a terminal diaminopropionate residue. We additionally developed HMMs and tailoring reactions for the N-sulfotransferase SulN (Monobactam_ST), the SulP O-methyltransferase, and the clavaminate synthase-like protein SulO, which is assumed to catalyze hydroxylation of a diaminopropionate ɑ-carbon; two separate HMMs were developed for phylogenetically distinct clades of SulO-family enzymes. Detection of putative sulfazecin-type BGCs requires the presence of the unique thioesterase, in addition to adenylation and thiolation domains. To provide information relating

to BGC detection, although not chemical structure prediction, for two other notable families of monobactams, we additionally developed models for the nocardicin C-9' epimerase NocJ and the tabtoxin β-lactam synthase TblS; detection of putative nocardicin-family BGCs requires NocJ and an adenylation domain, whereas detection of putative tabtoxin-family BGCs requires only TblS.

Penicillins and cephalosporins. Penicillins and cephalosporins are nonribosomal peptide-derived β-lactam antibiotics that share a cysteine-valine dipeptidyl core, at which the isopenicillin N synthase (IPNS), a selective oxygenase, catalyzes the formation of the β-lactam core. Isopenicillin N can be further derivatized by the isopenicillin N acyltransferase (IAT), which is assumed to replace the 2-amino adipic acid residue with a phenylacetate residue within PRISM. In cephalosporin family BGCs, the deacetoxycephalosporin synthase/hydroxylase (CefEF), for which two distinct HMMs were developed, further catalyzes ring expansion and subsequent hydroxylation of the remaining valine γ carbon to produce deacetylcephalosporin. Acetylation by the deacetylcephalosporin C acetyltransferase CefG, which is permitted to act at any free hydroxyl, produces cephalosporin C. In the carbamoyl-bearing cephamycin family, the deacetylcephalosporin precursor can instead be derivatized by the O-carbamoyltransferase CefG, the C-7 hydroxylase CmcJ, and the O-methyltransferase CmcI; CefG and CmcI are permitted to act at any free hydroxyl. Finally, we also constructed two models for the isopenicillin N epimerase CefD, although these are not associated with a virtual tailoring reaction as PRISM does not predict stereochemistry at this time. Detection of putative penicillin BGCs requires the presence of the IPNS and an adenylation domain, whereas cephalosporin-type BGCs additionally require CefEF and cephamycin-type BGCs require CmcH. The three classes are considered to be mutually exclusive within PRISM 4.

Lincosamides

Lincomycin A and celesticetin are the two major representatives of the family of lincosamide antibiotics, whose core structural features comprise an amino acid and an eight-carbon thiosugar. The former provides the basis for the clinically used antibiotic clindamycin, a semisynthetic chlorinated derivative. In lincomycin, the amino acid moiety consists of an N-methylated 4-propylproline residue, which is derived from tyrosine via a series of biosynthetic genes with homologs involved in hormaomycin and pyrrolobenzodiazepine biosynthesis. Celesticetin instead contains a proteinogenic proline residue, but additionally contains a salicylic acid residue linked to the thiosugar via a mercaptoethanol chain.

We developed hidden Markov models for lincosamide thiosugar biosynthesis, biosynthesis of 4-propylproline and related nonproteinogenic amino acids found in pyrrolobenzodiazepines and hormaomycin, and celesticetin salicylate attachment. Within PRISM, thiosugar biosynthesis begins with the activation of ribose-5-phosphate and fructose-6-phosphate by the transaldolase LmbR to form octulose-8-phosphate, followed by 1,2-isomerization by the LmbN isomerase to produce octose 8-phosphate[64]. Next, the LmbP kinase phosphorylates the C1 hydroxyl to yield octose-1,8-biphosphate, whereafter the LmbK phosphatase catalyzes hydrolysis to yield octose 1-phosphate[65]. The nucleotidyltransferase LmbO then catalyzes guanosine diphosphate (GDP) transfer to yield a nucleotide-activated octose; the thiosugar biosynthetic pathway continues with C6 amination and removal of the C8 hydroxyl group, but because the enzymes responsible for these transformations are unknown, these reactions are associated with LmbO instead of guanine diphosphate transfer. Installation of the C1 methylmercapto group proceeds with glycosyltransfer of the octose group onto the Actinomycete-specific low-molecular weight thiol ergothioneine by the LmbT S-glycosyltransferase, which is in turn followed by ligation of the 4-propylproline (or, in

celesticetin, proline) residue onto the C6 nitrogen by the unusual condensation protein LmbD[66]. The mycothioltransferase LmbV then catalyzes thiol exchange between ergothioneine and mycothiol, resulting in formation of a lincosamide–mycothiol conjugate, which is hydrolyzed by the mycothiol amidase LmbE, yielding an N-acetyl-cysteinyl lincosamide intermediate. After N-methylation of the proline or propylproline backbone nitrogen by LmbJ, the biosynthetic pathway of lincomycin and celesticetin branches. The lincomycin PLP-dependent protein LmbF catalyzes β-elimination to yield a sulfhydryl group. Alternatively, its homolog in celesticetin biosynthesis CcbF catalyzes a different reaction, with decarboxylation-dependent transamination yielding a mercaptoacetaldehyde moiety[67]. This is then reduced by the oxidoreductase Ccb5, and the lincosamide C7 hydroxyl is O-methylated by Ccb4[68]. Finally, the WS/DGAT acyltransferase Ccb1 and the salicylate-AMP ligase Ccb2 combine to transfer a salicylate moiety to the mercaptoethanol group[69]; within PRISM, the presence of either is assumed to be sufficient to catalyze this reaction, which is permitted to occur at any free hydroxyl. The lincomycin pathway terminates with the action of the S-methyltransferase LmbG to yield the methylmercapto sugar.

Biosynthesis of the 4-propylproline moiety of lincomycin begins with action of the unusual heme-containing tyrosine *ortho*-hydroxylase LmbB2, which is assumed within PRISM to activate and hydroxylate a tyrosine residue[70]. The L-DOPA glyoxylase LmbB1 then catalyzes 2,3-extradiol cleavage to form 2,3-*seco*dopa, with spontaneous cyclization to 4-(2-oxo-3-butenoic-acid)-4,5-dihydropyrrole-2-carboxylic acid[71]. The γ-glutamyltransferase LmbA next liberates oxalic acid via carbon–carbon bond cleavage[72], forming 4-vinyl-2,3-dihydropyrrole-2-carboxylic acid, a common intermediate in lincomycin, hormaomycin, and pyrrolobenzodiazepines. In tomaymycin, tautomerization by the 4-oxalocrotonate tautomerase TomN (or its phylogenetically distinct homolog Lim13) and reduction by the F_{420}-dependent oxidoreductase TomJ produce 4-ethylidene-tetrahydropyrrole-2-carboxylic acid[73]. In lincomycin, the C-methyltransferase LmbW catalyzes methylation of the vinyl group, followed by double bond tautomerization by the tautomerase LmbX (or any of its phylogenetically distinct homologs SibS, TomK, or Por16), and reduction by LmbY, to form 4-propylproline. In hormaomycin, the action of LmbX and LmbY is replaced by that of the F_{420}-dependent oxidoreductase HrmD 4-vinyl-2,3-dihydropyrrole-2-carboxylic acid intermediate, forming 4-propenylproline[74]. In pyrrolobenzodiazepines such as porothramycin and sibiromycin, the LmbY product is reduced by the F_{420}-dependent oxidoreductase SibT to produce 4-propylidene-tetrahydropyrrole-2-carboxylic acid, whereafter the FAD-dependent oxidoreductase SibW catalyzes the oxidation of the tetrahydropyrrole[75]. In pyrrolobenzodiazepines such as anthramycin and porothramycin, the pathway continues with allylic methyl oxidation of the propenyl side chain by the cytochrome P450 hydroxylase ORF4; sequential oxidation by the alcohol dehydrogenase ORF3 (or the phylogenetically distinct homolog Por23) and the aldehyde dehydrogenase ORF2; and amide formation by the transaminase ORF1 (Por8)[76]. Finally, in porothramycin the amide group is subsequently N,N-dimethylated by the Por25 N-methyltransferase.

Detection of putative lincosamide BGCs requires the presence of LmbT and one of either LmbF or CcbF. In light of the diversity of metabolites whose biosynthetic pathways involve common enzymatic machinery for alkylproline biosynthesis, we additionally designed a generic rule for alkylproline-containing BGCs, which requires the presence of LmbA and one of either LmbW or TomN.

Isonitrile alkaloids

We developed twelve new HMMs and eleven new virtual tailoring reactions to identify and predict complete chemical structures for isoprenoid-indole alkaloids, including members of the hapalindole, welwitindolinone, fisherindole and ambiguine families[77], among other metabolites[78,79]. The isonitrile synthase IsnA, which is often present in two copies these BGCs, is assumed to catalyze formation of the isonitrile group at a tryptophan or tyrosine residue. The α-ketoglutarate-dependent oxygenase IsnB then catalyzes double bond formation adjacent to the isonitrile and decarboxylation. An exception to this rule occurs if the chromophore-forming monooxygenase PvcC is present in the BGC, in which case the carboxyl group is retained and catechol formation, followed by intramolecular cyclization to form a coumarin, is predicted instead. The aromatic prenyltransferase FamD2 activates geranylpyrophosphate and catalyses indole geranylation, forming a quaternary carbon, which is subsequently rearomatized by one of four cyclases (FamC1, FamC2, FamC3, and FamC5) to form the hapalindole or fischerindole-type scaffold[80]. We also built HMMs and reactions for phylogenetically distinct clades of halogenases (AmbO5) and isoprenyltransferases (AmbP3) that further derivatize these scaffolds, with prenylation assumed to occur at the indole C2 and halogenation on any C1 of an isoprene group. Glycosyltransferases involved in tyrosine isonitrile biosynthesis (IsnGT) are assumed to preferentially glycosylate phenyl group C4 hydroxyls, but will act at any free hydroxyl in the molecule if one cannot be found, with their substrate assumed to be either glucose or N-acetylglucosamine. Finally, we built a HMM for the fischerindole-specific LuxR transcriptional regulator AmbR1.

Nucleosides

To capture the diversity of biosynthetic pathways for known nucleosides, we developed 145 HMMs and 133 virtual tailoring reactions to predict chemical structures for 21 different families of nucleosides. We review the HMMs and the reactions they catalyze in the context of the complete biosynthetic pathways for exemplary members of each family below. However, we emphasize that identification of every enzyme implicated in these biosynthetic pathways is not a prerequisite for accurate structure prediction. Of particular note are cases where many known members of a given family are distinguished by presence or absence of various tailoring enzymes, or combinations thereof, often acting at late stages in biosynthesis. The ability to naturally and flexibly account for this enzymatic diversification is a strength of the graph-based approach to structure prediction within PRISM.

Glycyluridine-derived nucleosides. This class of nucleosides are characterized by a biosynthetic pathway originating from a common 5’-C-glycyluridine precursor, with several subfamilies distinguished by their divergent courses of biosynthesis. Uridyl lipopeptides, including the liposidomycin- and muraymycin-type subfamilies, retain the 5’-C-glycyluridine moiety in their final structures and are additionally characterized structurally by the presence of an aminoribofuranoside. Of these, the liposidomycin-type subfamily, including caprazamycin, muraminomicin, and A-90289A, are further structurally characterized by a substituted diazepanone ring and variable fatty acid chains, whereas the muraymycin-type subfamily is instead linked via an aminopropyl group to the product of a nonribosomal peptide synthase. In contrast, the capuramycin-family nucleosides contain a uridine-5’-carboxamide moiety. PRISM implements a generic rule for glycyluridine-containing nucleoside BGC detection, requiring the presence of the uridine-5'-monophosphate dioxygenase LipL and the PLP-dependent uridine-5'-aldehyde transaldolase LipK, which together catalyze 5’-C-glycyluridine biosynthesis. More specific rules are also implemented for specific subfamilies as

follows. Liposidomycin-family nucleoside BGCs require the 5'-amino-5'-deoxyuridine phosphorylase LipP, the 3-amino-3-carboxypropyl aminotransferase Mur24, and the N-methyltransferase LipW in addition to LipK for detection. Muraymycin-family nucleosides require LipK, LipP, the PLP-dependent decarboxylase Mur23, and the unusual amide bond-forming β-lactamase[81] Mur30 for BGC detection. Capuramycin-family nucleosides require the α-mannopyranuronate transferase CapG and the N-transacylase CapW in addition to LipK for BGC detection.

The common biosynthetic pathway of glycyluridine-derived nucleosides begins with LipL, which activates uridine monophosphate (UMP) and converts it to uridine-5'-aldehyde. Because the aminoribose sugar found within the liposidomycin- and muraymycin-family nucleosides is also derived from uridine-5'-aldehyde, in BGCs that contain both LipK and LipP (implicated in 5'-C-glycyluridine and aminoribose biosynthesis, respectively), LipL is assumed to activate and react at two UMP residues; in clusters that contain LipL homologs but not both of LipK and LipP, such as capuramycin-family nucleosides, only one UMP residue is activated within PRISM. From uridine-5'-aldehyde, LipK activates a glycine residue and catalyzes 5'-C-glycyluridine synthesis, whereas the LipO aminotransferase catalyzes formation of 5'-amino-5'-deoxyuridine. This is followed by the action of the phosphorylase LipP, generating 5'-amino-5'-deoxy-α-ribose-1-phosphate, and the nucleoside nucleotidylyltransferase LipM, which catalyzes attachment of the aminosugar to uridine diphosphate; however, in order to minimize the combinatorial complexity within PRISM, the action of the LipM enzyme is modeled as simple removal of the phosphate group installed by LipP. The LipN glycosyltransferase then transfers the aminosugar to the 5'-C-glycyluridine residue at the 5' hydroxyl at to yield a disaccharide. Mur24 (LipJ) then transfers a methionine-derived 3-amino-3-carboxypropyl group to the glycine nitrogen, forming the last common intermediate of the liposidomycin-family and muraymycin-family pathways.

The liposidomycin pathway continues with the beta-hydroxylation of the methionine-derived group by LipG, followed by N,N-dimethylation and diazepanone ring formation by LipW. In reality, the enzyme responsible for diazepanone ring formation remains unknown; thus, the reaction is associated with LipW in PRISM. The pathway then terminates with acylation by LipT and LipR, which activate β-hydroxypalmitate and hydroxymethylglutarate respectively, and one of the LipB or LpmB sulfotransferases, which are sufficiently distinct in their primary sequences that separate HMMs were constructed. All four of these enzymes are assumed to potentially react at any free hydroxyl group, with the result that arbitrary combinations of acyl chains can be predicted, at the expense of a large combinatorial search space. In the muraymycin pathway, Mur23 decarboxylates the methionine-derived 3-amino-3-carboxypropyl group, and Mur30 catalyzes condensation with the nonribosomal peptide synthetase product at any carboxylic acid.

In the capuramycin-family pathway, the PLP-dependent monooxygenase[82] Cap15 catalyzes conversion of 5'-C-glycyluridine to uridine-5'-carboxamide. The CapG glycosyltransferase is then assumed to activate and transfer α-mannopyranuronate to any free hydroxyl. This reaction may be followed by O-methylation (CapK) at any free hydroxyl, carbamoylation (CapB) at any free hydroxyl, and/or carboxy-O-methylation (CapS) at any carboxyl group. The CapW N-transacylase catalyzes attachment of the carboxy-O-methyl group to the product of the nonribosomal peptide synthetase. To account for the formation of the aminocaprolactam group in several members of this family, we additionally built an HMM for the radical SAM C-methyltransferase CapT, which is associated with aminocaprolactam formation within PRISM in addition to ε-carbon methylation of a lysine residue.

We also constructed HMMs for several additional, subfamily-specific tailoring enzymes, including the liponucleoside rhamnose O-methyltransferases LipA1, LipY, and LipZ (permitted to act at any sugar 2'-hydroxyl, 3'-hydroxyl, and 4'-hydroxyl, respectively); the muraminomicin rhamnose

C6-methyltransferase Mra3 (permitted to at at any sugar C6); the liponucleoside rhamnosyltransferase LipB1, which follows the generic glycosyltransferase rules within PRISM; and the muraminomicin rhamnose O-3-succinyltransferase Mra4, which is permitted to acylate any free hydroxyl.

Blasticidin-, gougerotin-, and mildiomycin-family nucleosides. These three families of nucleosides are all characterized by a structural core consisting of a glucuronic acid-derived hexose sugar attached to a cytosine or hydroxymethylcytosine residue, but divergent biosynthesis at later stages. The shared biosynthetic pathway begins with the cytidine monophosphate (CMP) hydrolase BlsM, which catalyzes hydrolysis of CMP to yield cytidine. This is followed by the action of the cytosylglucuronic acid (CGA) synthase BlsD. Because some BGCs apparently lack homologs of BlsM, BlsD is assumed to activate both CMP and glucuronic acid. (In mildiomycin-family nucleosides, the CMP hydroxymethylase MilA first catalyzes the formation of hydroxymethyl-CMP.) The oxidoreductase GouA is then assumed to oxidize CGA to yield 4-carbonyl-CGA. Next, amination by the transaminase GouH, or one of its homologs, BlsH or MilD, for which distinct HMMs were constructed, yields 4-amino-CGA, the common intermediate for blasticidin-, gougerotin-, and mildiomycin-family nucleosides.

In gougerotin-family nucleosides, including yunnanmycin and ningnanmycin, the glycyl-CoA N-methyltransferase GouN activates and N-methylates a glycine residue, and the acyl-CoA synthetase GouK activates a serine or second glycine residue. The acyl-CoA N-acyltransferase GouJ is then assumed within PRISM to catalyze any possible permutation of condensation reactions between these activated amino acids and any pair of free nitrogens, each bonded to a sp^3 carbon.

In blasticidin- and mildiomycin-family nucleosides, the radical SAM enzyme MilG catalyzes dehydration of the glucuronic acid-derived moiety to form cytosinine. In blasticidin-family nucleosides, this is followed by ligation of an arginine-derived modified amino acid to the 4’-amino group, which is assumed within PRISM to be catalyzed by the lysyl-tRNA synthetase homolog ArgK (in theory, this is permitted to react at any sp^3 carbon-bonded free nitrogen within PRISM). Arginine can be activated by either the 2,3-aminomutase BlsG, forming β-arginine, or the aspartate aminotransferase ArgM. In arginomycin-type pathways, ArgM forms 5-guanidino-2-oxopentanoic acid, followed by C3-methylation by ArgN to form 5-guanidino-3-methyl-2-oxopentanoic acid, and iterative transamination by ArgM to yield β-methylarginine; for this reason, ArgM is not associated with a reaction within PRISM, but simply adds an arginine residue to the chemical graph. Finally, the guanidino methyltransferase ArgL catalyzes δ-N-methylation. We also built a HMM for the putative ligase ArgJ, although no reaction is included within PRISM.

In mildiomycin-family nucleosides, the aspartate aminotransferase MilM is assumed to convert arginine to α-keto-δ-guanidinovalerate. This is then assumed to be coupled to the gluruconic acid-derived moiety of the nucleoside by the dihydropicolinate synthetase homolog MilN. Finally, the ArgK homolog is assumed to activate serine and catalyze condensation onto a free sp^3 carbon-bonded nitrogen.

Octosyl acid-derived nucleosides. Nikkomycins and polyoxins are potent antifungal agents and exemplary members of a family of nucleosides structurally characterized by the presence of an aminohexuronic acid moiety derived from an octosyl acid precursor. They differ mainly in their peptidyl moieties, which contain variable numbers of highly modified nonproteinogenic amino acids. A generic rule for the detection of octosyl acid-derived nucleoside BGCs requires the presence of the UMP-enoylpyruvyltransferase PolA and the octosyl acid phosphate synthase PolH. BGC detection for

nucleosides of the polyoxin subfamily require the presence of the NADPH-dependent reductase PolM, the amide synthase PolG, and a protein with homology to either of paralogous α-ketoglutarate-dependent dioxygenases PolD/PolK, for which a single HMM was constructed, in addition to PolH. Nikkomycin-family BGCs require PolH, PolD/PolK, PolG, and the unique flavin-dependent oxidoreductase NikD.

The common biosynthetic pathway begins with the UMP-enoylpyruyltransferase PolA, which activates both uridine monophosphate and a pyruvate group to form 3'-enolpyruvyl-UMP. The radical SAM enzyme PolH then catalyzes rearrangement of 3'-enolpyruvyl-UMP to octosyl acid phosphate, followed by dephosphorylation by the PolJ phosphatase to produce octosyl acid. Two separate models were constructed for two phylogenetically distinct clades of PolJ homologs. The sequential action of PolD/PolK likely convert octosyl acid to ketohexuronic acid; since their respective functions have not been demonstrated experimentally, the presence of a homolog to either protein in a given BGC is considered sufficient to catalyze the reaction within PRISM. The PLP dependent aminotransferase PolI thereafter forms the aminohexuronic acid.

In some members of nikkomycin subfamily, the uracil moiety linked to the aminohexuronic acid is replaced by a 4-formyl-4-imidazolin-2-one base. This is derived from a histamine residue, which is activated by a nonribosomal synthetase (adenylation–thiolation didomain), then processed by the β-hydroxylase NikQ. The remainder of the biosynthetic pathway is not fully known, so is assumed within PRISM to be catalyzed by the phosphoribosyl transferase NikR, which is also assumed to catalyze replacement of the nucleobase.

Nikkomycin-family nucleosides are further characterized by a peptidic bond between the aminohexuronic acid and the unique nonproteinogenic acid hydroxypyridylhomothreonine (HPHT). Its biosynthesis begins with the lysine transaminase NikC, which catalyzes deamination to form an α-ketoacid, and then the unusual flavoprotein NikD, which catalyzes cyclization to picolinic acid. A standalone adenylation domain then activates picolinic acid to picolinate-CoA, for which a new substrate-specific HMM was developed (although this does not actually catalyze any reaction within PRISM). The acetaldehyde dehydrogenase NikA then converts picolinate-CoA to picolinaldehyde, and the aldolase NikU is assumed to catalyze condensation with 2-oxobutyrate to form 4-pyridyl-2-oxo-4-hydroxyisovalerate. Finally, the NikT acyl carrier protein/aminotransferase catalyzes transamination to form pyridylhomothreonine, and the NikF cytochrome P450 catalyzes hydroxylation to form HPHT. The nucleotidyltransferase PolG is assumed to catalyze amide bond formation to yield the assembled product.

In polyoxin-family nucleosides, the hydroxypyridylhomothreonine residue is replaced by carbamoylpolyoxamic acid (CPOAA), which is derived from glutamate. Its biosynthetic pathway begins with glutamate N-acetylation by PolN, followed by phosphorylation by the PolP kinase to form N-acetyl-γ-glutamyl phosphate and tandem reduction by the short chain dehydrogenase PolM to form α-acetamido-δ-hydroxyvaleric acid. At this point, PolN catalyzes deacetylation, so for simplicity within PRISM this domain is not associated with a tailoring reaction. The pathway proceeds with δ-carbamoylation by PolO and finally iterative hydroxylation by PolL to produce CPOAA. Polyoxins may also contain a polyoximic acid moiety, which is derived from isoleucine. Within PRISM, this is assumed to involve sequential dehydration by the oxidoreductase PolF to generate 2-amino-3-methyl-3-pentenoic acid, hydroxylation by the PolC hydroxylase to generate 2-amino-3-hydroxymethyl-3-pentenoic acid, and cyclization by PolE via an unknown mechanism. Finally, the PolG nucleotidyltransferase catalyzes one or two rounds of amide bond formation assembly of the final product.

Toyocamycin- and sangivamycin-family nucleosides. Toyocamycin and sangivamycin are exemplary members of the large family of pyrrolopyrimidine or deazapurine nucleosides, whose biosynthetic pathways are the best understood of any members of this family[83]. The earliest steps in the toyocamycin and sangivamycin biosynthetic pathways are shared with those in the biosynthesis of the modified nucleotide queuosine. A guanosine triphosphate (GTP) cyclohydrolase I protein (GCHI) activates GTP and catalyses its its conversion to 7,8-dihydroneopterin triphosphate, whereafter the 6-carboxy-5,6,7,8-tetrahydropterin synthase QueD, 7-carboxy-7-deazaguanine synthase QueE, and 7-cyano-7-deazaguanine synthase QueC act sequentially to produce 7-cyano-7-deazaguanine, the last common intermediate in both queuosine and deazapurine biosynthesis. Alternatively, in the tubercidin biosynthetic pathway, the UbiD family decarboxylase TubF catalyzes 7-carboxy-7-deazaguanine decarboxylation. To distinguish between clusters of genes involved in secondary metabolism and queuosine biosynthesis, we additionally developed a model for the nitrile oxidoreductase QueF, which catalyzes the NADPH-dependent conversion of 7-cyano-7-deazaguanine to 7-aminomethyl-7-deazaguanine (“preQ$_1$”). The presence of a QueF homolog in a candidate BGC is considered to violate the rules for detection of toyocamycin- and sangivamycin-family nucleosides.

Toyocamycin biosynthesis continues with the phosphoribosylpyrophosphate transferase ToyH (or its homolog TubE in tubercidin biosynthesis, for which a separate model was constructed), which glycosylates the purine base; the guanosine monophosphate (GMP) reductase ToyE, which catalyzes deamination of the purine base; and the adenylosuccinate synthetase/lyase pair ToyG and ToyF, which activate an aspartate residue and catalyze replacement of the ketone at C6 with a nitrogen group, releasing fumarate. Finally, the haloacid dehydrogenase superfamily enzyme ToyI (or its distinct homolog TubG in tubercidin biosynthesis) is then assumed to act as the phosphatase then dephosphorylates the toyocamycin-5’ monophosphate to yield toyocamycin. Toyocamycin can further be converted to sangivamycin by the action of the multi-subunit nitrile hydratase complex encoded by the ToyJKL genes, which catalyze hydration of the nitrile group to produce the corresponding amide. The presence of any one of these three subunits is considered sufficient to catalyze this reaction within PRISM. Detection of putative toyocamycin-family nucleoside BGCs requires the presence of ToyH (or TubE) and not QueF. Detection of sangivamycin-family BGCs additionally requires the presence of at least one of the ToyJKL nitrile hydratase subunits. The two subfamilies are considered to be mutually exclusive.

Pacidamycin-family nucleosides. This class of nucleosides, which also includes napsamycin and sansanmycin among other products, is characterized by the presence of a 3’-deoxy-4’,5’-enamino-uridine structural core. The common biosynthetic pathway begins with the flavin-dependent oxidoreductase Pac11, which activates a uridine residue and oxidizes it to uridine-5’-aldehyde. This is followed by the action of the aldo-uridine dehydratase Pac13, which catalyzes dehydration at the 3’ position, and the PLP-dependent aminotransferase Pac15, which catalyzes transamination at the 5’ position. Finally, the nucleotidyltransferase Pac9 catalyzes condensation of the modified nucleoside to the product of the nonribosomal peptide synthetase. This reaction is assumed to potentially occur at any carboxylic acid within PRISM. The presence of both Pac5 and Pac9 is required for detection of putative pacidamycin-family BGCs.

Tunicamycin-family nucleosides. This class of nucleosides, also called uridyl lipodisaccharide antibiotics, are characterized by a unique 11-carbon core, tunicamine, which is derivatized with uracil, N-acetylglucosamine, and variable fatty acid chains; the tunicamine core is also found in several other

products[84]. Detection of tunicamycin-family nucleoside BGCs requires the presence of the UDP-N-acetylglucosamine 4-epimerase TunF and the uridine-activating radical SAM enzyme TunB. The biosynthetic pathway begins with the activation of UDP-N-acetylglucosamine by TunF; because PRISM predictions do not include stereochemistry, the enzyme is associated only with subgraph activation and not with a tailoring reaction. Next, the short-chain dehydrogenase/reductase TunA catalyzes 5,6-dehydration of N-acetylglucosamine to form an exo-glycal intermediate. In parallel, TunB is assumed to activate a uridine residue and catalyze carbon–carbon bond formation to form UDP-N-acetyl-tunicamine. The pyrophosphatase TunH and deacetylase TunE then liberate UDP and an acetyl group, respectively, to form tunicamine. The final steps in the pathway involve tailoring by the TunD glycosyltransferase, which transfers a second N-acetylglucosamine to any free hydroxyl group, and the N-acyltranferase TunC, which transfers a cellular fatty acid (assumed within PRISM to be (E)-12-methyltridec-2-enoic acid) to any free nitrogen atom. Finally, we developed a model for the SAM-dependent methyltransferase TunM, which is putatively involved in tunicamycin resistance[85].

Puromycin-, hygromycin A-, and A201A-family nucleosides. These three classes of adenosyl nucleoside antibiotics share substantial portions of their biosynthetic machinery. The puromycin biosynthetic pathway begins with the action of the NAD-dependent adenosine triphosphate (ATP) dehydrogenase Pur10 and the aminotransferase Pur4 to catalyze the formation of 3'-amino-3'-deoxy-ATP; of these, Pur10 is specifically associated with the activation of an ATP residue within PRISM. These reactions are in turn followed by those catalyzed by the pyrophosphohydrolase Pur7 and the monophosphatase Pur3 to yield 3′-amino-3′-deoxyadenosine. The tyrosinyl-aminonucleoside synthetase Pur6 and the N-acetyltransferase Pac then activate a tyrosine residue and acetyl group, respectively, and catalyzes their attachment to any free nitrogen. A final series of tailoring reactions, including N^6,N^6-dimethylation of the adenine by the N-methyltranferase Pur5, removal of the acetyl group introduced by Pac by the deacetylase NapH, and O-methylation by DmpM (assumed within PRISM to potentially act at any hydroxyl group), produce puromycin. Detection of putative puromycin-family nucleoside BGCs requires the presence of Pur4, Pur5, and Pur10.

The structurally related antibiotic A201A shares several enzymes involved in puromycin biosynthesis[86]. As in puromycin biosynthesis, the action of Pur10, Pur4, Pur7, and Pur3 yields 3'-amino-3'-deoxy-adenosine, with Pur5 catalyzing adenine N^6,N^6-dimethylation. In parallel, a cassette of enzymes shared with hygromycin A biosynthesize and attach a *para*-hydroxy-α-methylcinnamic acid moiety at the 3'-amino position. First, the CoA ligase Hyg12 activates a *para*-hydroxybenzoic acid residue. The Hyg22 acyltransferase and Hyg10 β-ketoacyl synthase are then proposed to catalyze addition of a methylmalonyl group to form a 3-(4-hydroxyphenyl)-3-oxopropanoyl intermediate. The 3-keto group is then processed by the 3-ketoacyl ACP reductase Hyg15 and the 3-hydroxyacyl ACP dehydratase Hyg14 to generate the *para*-hydroxy-α-methylcinnamic acid moiety that is attached to the nucleoside. Because the enzyme responsible for amide bond formation is not known, the reaction is associated with the Hyg12 CoA ligase within PRISM, and assumed to potentially occur at any free nitrogen.

A201A-family nucleosides also contain a α-rhamnose moiety, which is shared with hygromycin A, and a unique unsaturated hexofuranose, which is not. The former is produced by short chain dehydrogenase/reductases MtdH and MtdJ, which catalyze the conversion of mannose to rhamnose via 4,6-dehydration. Within PRISM, MtdH is associated with the activation of a GDP-α-mannose residue. The glycosyltransferase MtdG1 then catalyzes both rhamnosylation at any free hydroxyl group and removal of GDP from the final structure. The epimerase MtdM activates a second

GDP-α-mannose residue and catalyzes its conversion to GDP-β-galactopyranose, whereafter the action of the GDP-L-galactose mutase MtdL generates GDP-galactofuranose. This is followed by 5'-O-methylation by the MtdM4 methyltransferase, C4/C5 dehydrogenation by MtdW, and finally transfer to the hydroxycinnamic acid moiety (in practice, any free hydroxyl within PRISM) and GDP elimination by the MtdtG2 glycosyltransferase. The final steps in A201A biosynthesis involve two O-methylations of the rhamnose moiety by the MtdM2 and MtdM3 O-methyltransferases, which are permitted to occur at any free hydroxyl group within PRISM.

In hygromycin A biosynthesis, the *para*-hydroxybenzoic acid-derived residue is replaced by a 3,4-dihydroxy-α-methylcinnamic acid moiety, whose biosynthetic pathway is distinguished from that in A201A biosynthesis by the action of the *para*-hydroxybenzoate hydroxylase Hyg2 on the *para*-hydroxybenzoic acid precursor. More significant is the presence of an aminocyclitol subunit, 2L-2-amino-2-deoxy-4,5-O-methylene-*neo*-inositol. The biosynthetic pathway for this moiety begins with the inositol dehydrogenase Hyg17, which activates a *myo*-inositol residue and catalyzes its conversion to 2-keto-*myo*-inositol. This is followed by the Hyg8 PLP-dependent aminotransferase, which yields *neo*-inosamine-2, and then 4'-O-methylation by the methyltransferase Hyg6. Finally, the metallo-dependent hydrolase Hyg7 catalyzes oxidative cyclization to form the methylenedioxy bridge. The final moiety of hygromycin A, a 5-dehydro-α-L-fucofuranose group, is produced from mannose via the sequential action of MtdH, MtdJ, MtdL, and finally the short-chain alcohol dehydrogenase Hyg20, which catalyzes oxidation at the 5' position. Detection of putative A201A-family nucleoside BGCs requires the presence of Hyg14 and MtdL, whereas detection of putative hygromycin A-family nucleoside BGCs additionally requires the presence of Hyg7.

Ascamycin-family nucleosides. Ascamycin and its analogues comprise a distinct family of adenosyl nucleoside antibiotics structurally characterized a unique 5'-O-sulfonamide moiety. Ascamycin biosynthesis is incompletely understood, but the sulfatase AcmG, acylsulfatase AcmI, and sulfotransferase AcmK are proposed to be involved in adenosine 5'-sulfonation[87]. Within PRISM, the presence of any of the three is considered sufficient to activate and sulfonylate an adenosine residue. The aminotransferase AcmN is then assumed to catalyze 5′-O-sulfonamide formation. In a similar manner, the flavin adenine dinucleotide (FAD)-dependent chlorinases AcmX and AcmY are thought to be involved in adenine C2-halogenation; the presence of either enzyme is considered sufficient to catalyze the reaction within PRISM. Finally, the AcmE esterase activates alanine and catalyzes conversion of dealanylascamycin to ascamycin, with homologs potentially acting at any free nitrogen. We additionally developed a model for the hypothetical protein AcmO to facilitate BGC identification under default PRISM parameters. Detection of putative ascamycin-family BGCs requires AcmN and any one of AcmGIK.

C-nucleosides. The nucleoside antibiotic showdomycin is a prototypical member of a family of atypical C-nucleosides, characterized by the presence of a carbon–carbon bond linking the nucleobase or nucleobase analog to the carbohydrate[88]. Although the biosynthesis of this nucleoside class remains poorly understood at present, we incorporated functionality for C-nucleoside BGC detection and limited structure prediction within PRISM 4. Detection of putative C-nucleoside BGCs requires the presence of the C-glycosynthase SdmA and the haloacid dehalogenase-superfamily phosphatase SdmB, which together catalyze C-glycosidic bond formation. Biosynthesis of the nucleobase analog is assumed to begin with the ectoine synthase homolog SdmE, which activates and cyclizes a glutamine residue. This is followed by deamination and ketone formation by the guanine deaminase homolog

SdmM, 3,4-desaturation by the FAD-dependent aryl-CoA dehydrogenase SdmF, and finally the formation of a C-nucleosidic bond to a ribose group activated by SdmA.

Amicetin-family nucleosides. Amicetin is a disaccharide pyrimidine nucleoside antibiotic with a unique mode of biosynthesis[89]. The AmiJ glycosyltransferase, a homlog of the blasticidin S cytosylglucuronic acid synthase BlsD, is assumed to catalyze N-glycosidic bond formation between amicetose and cytosine, with the AmiG glycosyltransferase further adding an amosamine or N-desmethyl-amosamine sugar at any free hydroxyl. The AmiF GNC5-related N-acetyltransferase then catalyzes amide bond formation between cytosine and *para*-aminobenzoic acid, which is provided by a homolog of the CmlB chorismate transaminase (this model having been developed during prediction of chloramphenicol biosynthesis). The AmiS hydroxymethyltransferase protein catalyzes α-methylserine formation from alanine, which is activated and transferred to a free nitrogen by the acyl-CoA-acyl carrier protein transacylase AmiR.

Jawsamycin-family nucleosides. Jawsamycin (FR-900848) is a structurally unique polycyclopropanated polyketide-nucleoside hybrid with antifungal activity. BGC detection requires the presence of the GCN5 family N-acetyltransferase homolog Jaw2 and the iterative cyclopropyl-forming radical SAM enzyme Jaw5[90]. Within the jawsamycin BGC, homologs of the glycyluridine-derived nucleoside enzymes LipL and LipO convert uridine-5’-monophosphate to uridine-5’-aldehyde and then 5’-amino-5’-deoxyuridine, respectively. The Jaw1 reductase then catalyzes reduction of the uracil moiety, and Jaw2 catalyzes condensation between the 5’-amino group of the nucleoside and the polyketide synthase product (in practice, any free carboxyl group). Because PRISM cannot predict iterative type I polyketides, a reaction could not be developed for the polycyclopropanation catalyzed by Jaw5.

References

1. Steinbeck, C. *et al.* Recent developments of the chemistry development kit (CDK) - an open-source java library for chemo- and bioinformatics. *Curr. Pharm. Des.* **12,** 2111–2120 (2006).
2. Prlić, A. *et al.* BioJava: an open-source framework for bioinformatics in 2012. *Bioinformatics* **28,** 2693–2695 (2012).
3. Camacho, C. *et al.* BLAST+: architecture and applications. *BMC Bioinformatics* **10,** 421 (2009).
4. Eddy, S. R. Accelerated profile HMM searches. *PLoS Comput. Biol.* **7,** e1002195 (2011).
5. Grant, C. E., Bailey, T. L. & Noble, W. S. FIMO: scanning for occurrences of a given motif. *Bioinformatics* **27,** 1017–1018 (2011).
6. Skinnider, M. A. *et al.* Genomic charting of ribosomally synthesized natural product chemical space facilitates targeted mining. *Proc Natl Acad Sci USA* **113,** E6343–E6351 (2016).
7. Hyatt, D. *et al.* Prodigal: prokaryotic gene recognition and translation initiation site identification. *BMC Bioinformatics* **11,** 119 (2010).
8. Edgar, R. C. MUSCLE: multiple sequence alignment with high accuracy and high throughput. *Nucleic Acids Res.* **32,** 1792–1797 (2004).
9. Skinnider, M. A., Johnston, C. W., Merwin, N. J., Dejong, C. A. & Magarvey, N. A. Global analysis of prokaryotic tRNA-derived cyclodipeptide biosynthesis. *BMC Genomics* **19,** 45 (2018).
10. Johnston, C. W. *et al.* An automated Genomes-to-Natural Products platform (GNP) for the discovery of modular natural products. *Nat. Commun.* **6,** 8421 (2015).
11. Skinnider, M. A. *et al.* Genomes to natural products PRediction Informatics for Secondary Metabolomes (PRISM). *Nucleic Acids Res.* **43,** 9645–9662 (2015).
12. Khayatt, B. I., Overmars, L., Siezen, R. J. & Francke, C. Classification of the adenylation and acyl-transferase activity of NRPS and PKS systems using ensembles of substrate specific hidden Markov models. *PLoS ONE* **8,** e62136 (2013).
13. Ziemert, N. *et al.* The natural product domain seeker NaPDoS: a phylogeny based bioinformatic tool to classify secondary metabolite gene diversity. *PLoS ONE* **7,** e34064 (2012).
14. Rausch, C., Hoof, I., Weber, T., Wohlleben, W. & Huson, D. H. Phylogenetic analysis of condensation domains in NRPS sheds light on their functional evolution. *BMC Evol. Biol.* **7,** 78 (2007).
15. Jacques, I. B. *et al.* Analysis of 51 cyclodipeptide synthases reveals the basis for substrate specificity. *Nat. Chem. Biol.* **11,** 721–727 (2015).
16. Bisang, C. *et al.* A chain initiation factor common to both modular and aromatic polyketide synthases. *Nature* **401,** 502–505 (1999).
17. Bretschneider, T. *et al.* Vinylogous chain branching catalysed by a dedicated polyketide synthase module. *Nature* **502,** 124–128 (2013).
18. Gu, L. *et al.* GNAT-like strategy for polyketide chain initiation. *Science* **318,** 970–974 (2007).
19. Chan, Y. A. *et al.* Hydroxymalonyl-acyl carrier protein (ACP) and aminomalonyl-ACP are two additional type I polyketide synthase extender units. *Proc Natl Acad Sci USA* **103,** 14349–14354 (2006).
20. Lim, S.-K. *et al.* iso-Migrastatin, migrastatin, and dorrigocin production in Streptomyces platensis NRRL 18993 is governed by a single biosynthetic machinery featuring an acyltransferase-less type I polyketide synthase. *J. Biol. Chem.* **284,** 29746–29756 (2009).
21. Helfrich, E. J. N. & Piel, J. Biosynthesis of polyketides by trans-AT polyketide synthases. *Nat. Prod. Rep.* **33,** 231–316 (2016).
22. Miyanaga, A., Kudo, F. & Eguchi, T. Mechanisms of β-amino acid incorporation in polyketide

macrolactam biosynthesis. *Curr. Opin. Chem. Biol.* **35,** 58–64 (2016).
23. Garneau, S., Dorrestein, P. C., Kelleher, N. L. & Walsh, C. T. Characterization of the formation of the pyrrole moiety during clorobiocin and coumermycin A1 biosynthesis. *Biochemistry* **44,** 2770–2780 (2005).
24. Ishida, K., Fritzsche, K. & Hertweck, C. Geminal tandem C-methylation in the discoid resistomycin pathway. *J. Am. Chem. Soc.* **129,** 12648–12649 (2007).
25. Fritzsche, K., Ishida, K. & Hertweck, C. Orchestration of discoid polyketide cyclization in the resistomycin pathway. *J. Am. Chem. Soc.* **130,** 8307–8316 (2008).
26. Skinnider, M. A., Merwin, N. J., Johnston, C. W. & Magarvey, N. A. PRISM 3: expanded prediction of natural product chemical structures from microbial genomes. *Nucleic Acids Res.* **45,** W49–W54 (2017).
27. Thibodeaux, C. J., Melançon, C. E. & Liu, H. Natural-product sugar biosynthesis and enzymatic glycodiversification. *Angew Chem Int Ed Engl* **47,** 9814–9859 (2008).
28. Kersten, R. D. *et al.* Glycogenomics as a mass spectrometry-guided genome-mining method for microbial glycosylated molecules. *Proc Natl Acad Sci USA* **110,** E4407-16 (2013).
29. Ichinose, K., Ozawa, M., Itou, K., Kunieda, K. & Ebizuka, Y. Cloning, sequencing and heterologous expression of the medermycin biosynthetic gene cluster of Streptomyces sp. AM-7161: towards comparative analysis of the benzoisochromanequinone gene clusters. *Microbiology (Reading, Engl)* **149,** 1633–1645 (2003).
30. Koketsu, K., Watanabe, K., Suda, H., Oguri, H. & Oikawa, H. Reconstruction of the saframycin core scaffold defines dual Pictet-Spengler mechanisms. *Nat. Chem. Biol.* **6,** 408–410 (2010).
31. Amagai, K., Takaku, R., Kudo, F. & Eguchi, T. A unique amino transfer mechanism for constructing the β-amino fatty acid starter unit in the biosynthesis of the macrolactam antibiotic cremimycin. *Chembiochem* **14,** 1998–2006 (2013).
32. Kudo, F. *et al.* Genome mining of the hitachimycin biosynthetic gene cluster: involvement of a phenylalanine-2,3-aminomutase in biosynthesis. *Chembiochem* **16,** 909–914 (2015).
33. Hotta, K. *et al.* Enzymatic catalysis of anti-Baldwin ring closure in polyether biosynthesis. *Nature* **483,** 355–358 (2012).
34. Luhavaya, H. *et al.* Enzymology of pyran ring a formation in salinomycin biosynthesis. *Angew Chem Int Ed Engl* **54,** 13622–13625 (2015).
35. Johnston, C. W., Zvanych, R., Khyzha, N. & Magarvey, N. A. Nonribosomal assembly of natural lipocyclocarbamate lipoprotein-associated phospholipase inhibitors. *Chembiochem* **14,** 431–435 (2013).
36. Schimming, O. *et al.* Structure, biosynthesis, and occurrence of bacterial pyrrolizidine alkaloids. *Angew Chem Int Ed Engl* **54,** 12702–12705 (2015).
37. Hari, T. P. A., Labana, P., Boileau, M. & Boddy, C. N. An evolutionary model encompassing substrate specificity and reactivity of type I polyketide synthase thioesterases. *Chembiochem* **15,** 2656–2661 (2014).
38. Gui, C. *et al.* Discovery of a new family of Dieckmann cyclases essential to tetramic acid and pyridone-based natural products biosynthesis. *Org. Lett.* **17,** 628–631 (2015).
39. Cociancich, S. *et al.* The gyrase inhibitor albicidin consists of p-aminobenzoic acids and cyanoalanine. *Nat. Chem. Biol.* **11,** 195–197 (2015).
40. Johnston, C. W. & Magarvey, N. A. Natural products: untwisting the antibiotic'ome. *Nat. Chem. Biol.* **11,** 177–178 (2015).
41. Schultz, A. W. *et al.* Biosynthesis and structures of cyclomarins and cyclomarazines, prenylated cyclic peptides of marine actinobacterial origin. *J. Am. Chem. Soc.* **130,** 4507–4516 (2008).

42. Du, Y.-L., He, H.-Y., Higgins, M. A. & Ryan, K. S. A heme-dependent enzyme forms the nitrogen-nitrogen bond in piperazate. *Nat. Chem. Biol.* **13,** 836–838 (2017).
43. Luesch, H. *et al.* Biosynthesis of 4-methylproline in cyanobacteria: cloning of nosE and nosF genes and biochemical characterization of the encoded dehydrogenase and reductase activities. *J. Org. Chem.* **68,** 83–91 (2003).
44. Goering, A. W. *et al.* Metabologenomics: Correlation of Microbial Gene Clusters with Metabolites Drives Discovery of a Nonribosomal Peptide with an Unusual Amino Acid Monomer. *ACS Cent. Sci.* **2,** 99–108 (2016).
45. Hasebe, F. *et al.* Amino-group carrier-protein-mediated secondary metabolite biosynthesis in Streptomyces. *Nat. Chem. Biol.* **12,** 967–972 (2016).
46. Liu, Y. *et al.* Identification and characterization of the ficellomycin biosynthesis gene cluster from Streptomyces ficellus. *Appl. Microbiol. Biotechnol.* **101,** 7589–7602 (2017).
47. Mahenthiralingam, E. *et al.* Enacyloxins are products of an unusual hybrid modular polyketide synthase encoded by a cryptic Burkholderia ambifaria Genomic Island. *Chem. Biol.* **18,** 665–677 (2011).
48. Ishida, K. *et al.* Biosynthesis and structure of aeruginoside 126A and 126B, cyanobacterial peptide glycosides bearing a 2-carboxy-6-hydroxyoctahydroindole moiety. *Chem. Biol.* **14,** 565–576 (2007).
49. Muliandi, A. *et al.* Biosynthesis of the 4-methyloxazoline-containing nonribosomal peptides, JBIR-34 and -35, in Streptomyces sp. Sp080513GE-23. *Chem. Biol.* **21,** 923–934 (2014).
50. Morinaka, B. I. *et al.* Natural noncanonical protein splicing yields products with diverse β-amino acid residues. *Science* **359,** 779–782 (2018).
51. Metlitskaya, A. *et al.* Maturation of the translation inhibitor microcin C. *J. Bacteriol.* **191,** 2380–2387 (2009).
52. Tsibulskaya, D. *et al.* The Product of Yersinia pseudotuberculosis mcc Operon Is a Peptide-Cytidine Antibiotic Activated Inside Producing Cells by the TldD/E Protease. *J. Am. Chem. Soc.* **139,** 16178–16187 (2017).
53. Ghodge, S. V., Biernat, K. A., Bassett, S. J., Redinbo, M. R. & Bowers, A. A. Post-translational Claisen Condensation and Decarboxylation en Route to the Bicyclic Core of Pantocin A. *J. Am. Chem. Soc.* **138,** 5487–5490 (2016).
54. Frattaruolo, L., Lacret, R., Cappello, A. R. & Truman, A. W. A Genomics-Based Approach Identifies a Thioviridamide-Like Compound with Selective Anticancer Activity. *ACS Chem. Biol.* **12,** 2815–2822 (2017).
55. Wang, H. *et al.* The glycosyltransferase involved in thurandacin biosynthesis catalyzes both O- and S-glycosylation. *J. Am. Chem. Soc.* **136,** 84–87 (2014).
56. Kudo, F. & Eguchi, T. Biosynthetic genes for aminoglycoside antibiotics. *J. Antibiot.* **62,** 471–481 (2009).
57. Caines, M. E. C., Elkins, J. M., Hewitson, K. S. & Schofield, C. J. Crystal structure and mechanistic implications of N2-(2-carboxyethyl)arginine synthase, the first enzyme in the clavulanic acid biosynthesis pathway. *J. Biol. Chem.* **279,** 5685–5692 (2004).
58. Bachmann, B. O., Li, R. & Townsend, C. A. beta-Lactam synthetase: a new biosynthetic enzyme. *Proc Natl Acad Sci USA* **95,** 9082–9086 (1998).
59. Zhang, Z. *et al.* Structural origins of the selectivity of the trifunctional oxygenase clavaminic acid synthase. *Nat. Struct. Biol.* **7,** 127–133 (2000).
60. Mosher, R. H., Paradkar, A. S., Anders, C., Barton, B. & Jensen, S. E. Genes specific for the biosynthesis of clavam metabolites antipodal to clavulanic acid are clustered with the gene for

clavaminate synthase 1 in Streptomyces clavuligerus. *Antimicrob. Agents Chemother.* **43,** 1215–1224 (1999).

61. Sleeman, M. C. & Schofield, C. J. Carboxymethylproline synthase (CarB), an unusual carbon-carbon bond-forming enzyme of the crotonase superfamily involved in carbapenem biosynthesis. *J. Biol. Chem.* **279,** 6730–6736 (2004).
62. Marous, D. R. *et al.* Consecutive radical S-adenosylmethionine methylations form the ethyl side chain in thienamycin biosynthesis. *Proc Natl Acad Sci USA* **112,** 10354–10358 (2015).
63. Oliver, R. A., Li, R. & Townsend, C. A. Monobactam formation in sulfazecin by a nonribosomal peptide synthetase thioesterase. *Nat. Chem. Biol.* **14,** 5–7 (2018).
64. Sasaki, E., Lin, C.-I., Lin, K.-Y. & Liu, H.-W. Construction of the octose 8-phosphate intermediate in lincomycin A biosynthesis: characterization of the reactions catalyzed by LmbR and LmbN. *J. Am. Chem. Soc.* **134,** 17432–17435 (2012).
65. Lin, C.-I., Sasaki, E., Zhong, A. & Liu, H. In vitro characterization of LmbK and LmbO: identification of GDP-D-erythro-α-D-gluco-octose as a key intermediate in lincomycin A biosynthesis. *J. Am. Chem. Soc.* **136,** 906–909 (2014).
66. Zhao, Q., Wang, M., Xu, D., Zhang, Q. & Liu, W. Metabolic coupling of two small-molecule thiols programs the biosynthesis of lincomycin A. *Nature* **518,** 115–119 (2015).
67. Kamenik, Z. *et al.* Deacetylation of mycothiol-derived “waste product” triggers the last biosynthetic steps of lincosamide antibiotics. *Chem. Sci.* **7,** 430–435 (2016).
68. Wang, M., Zhao, Q., Zhang, Q. & Liu, W. Differences in PLP-Dependent Cysteinyl Processing Lead to Diverse S-Functionalization of Lincosamide Antibiotics. *J. Am. Chem. Soc.* **138,** 6348–6351 (2016).
69. Kadlcik, S., Kamenik, Z., Vasek, D., Nedved, M. & Janata, J. Elucidation of salicylate attachment in celesticetin biosynthesis opens the door to create a library of more efficient hybrid lincosamide antibiotics. *Chem. Sci.* **8,** 3349–3355 (2017).
70. Novotna, J. *et al.* Lincomycin biosynthesis involves a tyrosine hydroxylating heme protein of an unusual enzyme family. *PLoS ONE* **8,** e79974 (2013).
71. Saha, S., Li, W., Gerratana, B. & Rokita, S. E. Identification of the dioxygenase-generated intermediate formed during biosynthesis of the dihydropyrrole moiety common to anthramycin and sibiromycin. *Bioorg. Med. Chem.* **23,** 449–454 (2015).
72. Zhong, G., Zhao, Q., Zhang, Q. & Liu, W. 4-alkyl-L-(Dehydro)proline biosynthesis in actinobacteria involves N-terminal nucleophile-hydrolase activity of γ-glutamyltranspeptidase homolog for C-C bond cleavage. *Nat. Commun.* **8,** 16109 (2017).
73. Li, W., Chou, S., Khullar, A. & Gerratana, B. Cloning and characterization of the biosynthetic gene cluster for tomaymycin, an SJG-136 monomeric analog. *Appl. Environ. Microbiol.* **75,** 2958–2963 (2009).
74. Cai, X. *et al.* Manipulation of regulatory genes reveals complexity and fidelity in hormaomycin biosynthesis. *Chem. Biol.* **20,** 839–846 (2013).
75. Li, W., Khullar, A., Chou, S., Sacramo, A. & Gerratana, B. Biosynthesis of sibiromycin, a potent antitumor antibiotic. *Appl. Environ. Microbiol.* **75,** 2869–2878 (2009).
76. Hu, Y. *et al.* Benzodiazepine biosynthesis in Streptomyces refuineus. *Chem. Biol.* **14,** 691–701 (2007).
77. Micallef, M. L. *et al.* Comparative analysis of hapalindole, ambiguine and welwitindolinone gene clusters and reconstitution of indole-isonitrile biosynthesis from cyanobacteria. *BMC Microbiol.* **14,** 213 (2014).
78. Clarke-Pearson, M. F. & Brady, S. F. Paerucumarin, a new metabolite produced by the pvc gene

cluster from Pseudomonas aeruginosa. *J. Bacteriol.* **190,** 6927–6930 (2008).
79. Crawford, J. M., Portmann, C., Zhang, X., Roeffaers, M. B. J. & Clardy, J. Small molecule perimeter defense in entomopathogenic bacteria. *Proc Natl Acad Sci USA* **109,** 10821–10826 (2012).
80. Li, S. *et al.* Decoding cyclase-dependent assembly of hapalindole and fischerindole alkaloids. *Nat. Chem. Biol.* **13,** 467–469 (2017).
81. Funabashi, M. *et al.* An ATP-independent strategy for amide bond formation in antibiotic biosynthesis. *Nat. Chem. Biol.* **6,** 581–586 (2010).
82. Huang, Y. *et al.* Pyridoxal-5'-phosphate as an oxygenase cofactor: Discovery of a carboxamide-forming, α-amino acid monooxygenase-decarboxylase. *Proc Natl Acad Sci USA* **115,** 974–979 (2018).
83. McCarty, R. M. & Bandarian, V. Deciphering deazapurine biosynthesis: pathway for pyrrolopyrimidine nucleosides toyocamycin and sangivamycin. *Chem. Biol.* **15,** 790–798 (2008).
84. Wyszynski, F. J. *et al.* Biosynthesis of the tunicamycin antibiotics proceeds via unique exo-glycal intermediates. *Nat. Chem.* **4,** 539–546 (2012).
85. Widdick, D. *et al.* Analysis of the Tunicamycin Biosynthetic Gene Cluster of Streptomyces chartreusis Reveals New Insights into Tunicamycin Production and Immunity. *Antimicrob. Agents Chemother.* **62,** (2018).
86. Zhu, Q. *et al.* Deciphering the sugar biosynthetic pathway and tailoring steps of nucleoside antibiotic A201A unveils a GDP-l-galactose mutase. *Proc Natl Acad Sci USA* **114,** 4948–4953 (2017).
87. Zhao, C. *et al.* Characterization of biosynthetic genes of ascamycin/dealanylascamycin featuring a 5'-O-sulfonamide moiety in Streptomyces sp. JCM9888. *PLoS ONE* **9,** e114722 (2014).
88. Palmu, K. *et al.* Discovery of the Showdomycin Gene Cluster from Streptomyces showdoensis ATCC 15227 Yields Insight into the Biosynthetic Logic of C-Nucleoside Antibiotics. *ACS Chem. Biol.* **12,** 1472–1477 (2017).
89. Zhang, G. *et al.* Characterization of the amicetin biosynthesis gene cluster from Streptomyces vinaceusdrappus NRRL 2363 implicates two alternative strategies for amide bond formation. *Appl. Environ. Microbiol.* **78,** 2393–2401 (2012).
90. Hiratsuka, T. *et al.* Biosynthesis of the structurally unique polycyclopropanated polyketide-nucleoside hybrid jawsamycin (FR-900848). *Angew Chem Int Ed Engl* **53,** 5423–5426 (2014).
