# Supplementary material for: Comprehensive prediction of secondary metabolite structure and biological activity from microbial genome sequences
Source: Nat Commun. 2020 Nov 27;11:6058. doi: 10.1038/s41467-020-19986-1 (PMC7699628; doi:10.1038/s41467-020-19986-1)
Supplement: Supplementary file 3 — Description of Additional Supplementary Files [file 41467_2020_19986_MOESM3_ESM.pdf]

Description of Additional Supplementary Files

File Name: Supplementary Data 1
Description: Complete catalog of biosynthetic domains (a), enzymatic tailoring reactions (b), substrates (c), and sugars (d) comprising the PRISM 4 chemical structure prediction engine.

File Name: Supplementary Data 2
Description: Structures of known products and predicted chemical structures, and Tanimoto coefficients between them, for the 1,281 characterized BGCs in the 'gold standard' set.

File Name: Supplementary Data 3
Description: Predicted chemical structures from PRISM 4 and antiSMASH 5 for BGCs identified in 3,759 complete bacterial genomes (a) and 6,362 metagenome-associated genomes (b).
