# Supplementary material for: Comprehensive prediction of secondary metabolite structure and biological activity from microbial genome sequences
Source: Nat Commun. 2020 Nov 27;11:6058. doi: 10.1038/s41467-020-19986-1 (PMC7699628; doi:10.1038/s41467-020-19986-1)
Supplement: Supplementary file 7 — Reporting Summary [file 41467_2020_19986_MOESM7_ESM.pdf]

nature research

Corresponding author(s): Michael A. Skinnider, Chris A. Dejong

Last updated by author(s): October 20, 2020

Reporting Summary

Nature Research wishes to improve the reproducibility of the work that we publish. This form provides structure for consistency and transparency in reporting. For further information on Nature Research policies, see Authors & Referees and the Editorial Policy Checklist.

Statistics

For all statistical analyses, confirm that the following items are present in the figure legend, table legend, main text, or Methods section.

n/a	Confirmed	
☐	☒	The exact sample size (*n*) for each experimental group/condition, given as a discrete number and unit of measurement
☒	☐	A statement on whether measurements were taken from distinct samples or whether the same sample was measured repeatedly
☐	☒	The statistical test(s) used AND whether they are one- or two-sided<br>*Only common tests should be described solely by name; describe more complex techniques in the Methods section.*
☒	☐	A description of all covariates tested
☐	☒	A description of any assumptions or corrections, such as tests of normality and adjustment for multiple comparisons
☐	☒	A full description of the statistical parameters including central tendency (e.g. means) or other basic estimates (e.g. regression coefficient) AND variation (e.g. standard deviation) or associated estimates of uncertainty (e.g. confidence intervals)
☐	☒	For null hypothesis testing, the test statistic (e.g. *F*, *t*, *r*) with confidence intervals, effect sizes, degrees of freedom and *P* value noted<br>*Give P values as exact values whenever suitable.*
☒	☐	For Bayesian analysis, information on the choice of priors and Markov chain Monte Carlo settings
☒	☐	For hierarchical and complex designs, identification of the appropriate level for tests and full reporting of outcomes
☒	☐	Estimates of effect sizes (e.g. Cohen's *d*, Pearson's *r*), indicating how they were calculated

Our web collection on statistics for biologists contains articles on many of the points above.

Software and code

Policy information about availability of computer code

Data collection	Chemical structure predictions were generated with PRISM (version 4.3.5) and antiSMASH (version 5.1.2). Raw outputs from PRISM and antiSMASH were processed using python (version 3.7.4). Code developed in this study is available from https://github.com/adapsyn/prism-4-paper.
Data analysis	Statistical analyses were performed in R (version 3.5.2), using the `nparcomp` (version 2.6), `AUC` (version 0.3.0), `pROC` (version 1.14.0) packages. Other aspects of data analysis were performed with the `jsonlite` (version 1.6), `magrittr` (version 1.5), `tidyverse` (version 1.2.1), and `uwot` (version 0.0.0.9010) packages. Plotting was performed with `ggplot2` (version 3.1.0) and `patchwork` (version 0.0.1). PRISM 4 is a Java 7 web application, freely available as an online service for the research community at http://prism.adapsyn.com. The PRISM web application is powered by Vue.js with a lightweight Python Flask API using PostgreSQL and Redis for queue management, providing a scalable solution that can process many submissions at once. PRISM 4 implements the Chemistry Development Kit (version 1.4.19) for chemical structure prediction and all in silico tailoring reactions, and BioJava (version 4.2.9)2 for some sequence file input and output operations. Other Java library dependencies include Apache Batik, Apache Commons, and Apache HttpComponents; ‘combinatoricslib’ (version 2.0, available from https://github.com/dpaukov/combinatoricslib); and the Jackson JSON processing library. System dependencies include BLAST+ (version 2.2.30) and HMMER (version 3.1b2) for protein similarity search. Optional system dependencies include FIMO (version 4.11.1), for RiPP precursor peptide cleavage6; Prodigal (version 2.6.1), for prokaryotic ORF prediction; MUSCLE (version 3.8.31), for active site residue identification in tRNA-derived cyclodipeptide synthases (CDPSs); and R (version 3.3.1), with packages ‘class’ (version 7.3-12), ‘e1071’ (version 1.6-7), and ‘plyr’ (version 1.8.4), for CDPS aminoacyl-tRNA substrate prediction.

For manuscripts utilizing custom algorithms or software that are central to the research but not yet described in published literature, software must be made available to editors/reviewers. We strongly encourage code deposition in a community repository (e.g. GitHub). See the Nature Research guidelines for submitting code & software for further information.

Data

Policy information about availability of data

All manuscripts must include a data availability statement. This statement should provide the following information, where applicable:

- Accession codes, unique identifiers, or web links for publicly available datasets
- A list of figures that have associated raw data
- A description of any restrictions on data availability

The genomes analyzed in this study are publicly available from the NCBI Genome database and the Sequence Read Archive (accession PRJNA348753). Predicted and true chemical structures from the 'gold standard' set of 1,281 BGCs are provided in Supplementary Data 2. Predicted chemical structures from the collection of 10,121 complete or metagenome-assembled prokaryotic genomes analyzed in this study are provided in Supplementary Data 3. FASTA files for the 'gold standard' BGCs are available via Zenodo (https://doi.org/10.5281/zenodo.3985982). PRISM output files, in JSON format, for all of the genomes analyzed in this study are available via Zenodo (https://doi.org/10.10.5281/zenodo.3985978). Links to other databases used include MIBiG (https://mibig.secondarymetabolites.org/), ClusterMine360 (http://clustermine360.ca/), and NRPS-PKS (http://202.54.226.228/~pksdb/sbspks_updated/search_main_pks_nrps.html). DoBISCUIT is no longer publicly available (notice at https://www.nite.go.jp/nbrc/information/20190325.html).

Field-specific reporting

Please select the one below that is the best fit for your research. If you are not sure, read the appropriate sections before making your selection.

☒ Life sciences ☐ Behavioural & social sciences ☐ Ecological, evolutionary & environmental sciences

For a reference copy of the document with all sections, see nature.com/documents/nr-reporting-summary-flat.pdf

Life sciences study design

All studies must disclose on these points even when the disclosure is negative.

Sample size	The sample size for the analyses of the 'gold standard' set of 1,281 BGCs presented in Fig. 2 was determined by the number of BGCs with fully characterized products that we could identify after an exhaustive review of the literature and existing databases. The sample size for the analysis of 3,759 dereplicated complete bacterial genomes was determined by the data publicly available in the NCBI Genome repository and the dereplication procedure described by Parks et al. (ref. 15). The sample size for the analysis of 6,362 dereplicated metagenome-assembled genomes (MAGs) was determined by the authors of the original study (ref. 23) using the same dereplication procedure.
Data exclusions	No data were excluded from the analyses.
Replication	Physicochemical and structural trends in predicted chemical structures were successfully reproduced in a set of 3,759 dereplicated complete bacterial genomese and an independent set of 6,362 dereplicated metagenome-assembled genomes.
Randomization	Randomization was not relevant to the study because analyses involved comparisons of computational methods, which were each applied to identical inputs.
Blinding	During the manual review of PRISM-only and antiSMASH-only BGCs, investigators were blinded to which software package had detected the BGC in question.

Reporting for specific materials, systems and methods

We require information from authors about some types of materials, experimental systems and methods used in many studies. Here, indicate whether each material, system or method listed is relevant to your study. If you are not sure if a list item applies to your research, read the appropriate section before selecting a response.

Materials & experimental systems

n/a	Involved in the study	
☒	☐	Antibodies
☒	☐	Eukaryotic cell lines
☒	☐	Palaeontology
☒	☐	Animals and other organisms
☒	☐	Human research participants
☒	☐	Clinical data

Methods

n/a	Involved in the study	
☒	☐	ChIP-seq
☒	☐	Flow cytometry
☒	☐	MRI-based neuroimaging
